# Too Many Doors, and Too Few: Perceived Career Option Breadth, Anticipated Regret and Career Decision-Making Difficulties in a Three-Wave Study of Chinese Young Adults

**DOI:** 10.3390/bs16101826

**Published:** 2026-10-06

**Authors:** Xinyu Cai, Tiantian Mo

**Affiliations:** College of Business, Jiaxing University, Jiaxing 314001, China

**Keywords:** career decision-making difficulties, choice overload, anticipated regret, maximizing tendency, option breadth, youth career decisions, time-lagged design

## Abstract

Career guidance usually treats a narrow option set as the problem, and career decision-making difficulties are modeled as decreasing in the range of options a young person perceives. This study asked whether that association is monotone. Three questionnaires spaced four weeks apart were completed by 1036 Chinese young adults (mean age 23.7) making a career decision: perceived career option breadth, maximizing tendency and career decision-making self-efficacy at Wave 1, anticipated career regret at Wave 2, and career decision-making difficulties at Wave 3. The data provided evidence of a U shape (quadratic *b* = 0.089, *p* < 0.001; Lind–Mehlum test *p* = 0.004). Difficulties reached a minimum 0.66 standard deviations above the mean (about 6.5 occupations in a parallel count-based model); the rise beyond it was less precisely estimated and sensitive to trimming and to the test used. Anticipated career regret rose convexly with option breadth, and accounting for it reduced the quadratic coefficient by 78%, while the direct association remained negative. Participants with a stronger maximizing tendency reached the estimated turning point at a lower option breadth. Career decision-making self-efficacy did not significantly weaken the regret–difficulty association. Wider option sets were associated with fewer difficulties only up to a point.

## 1. Introduction

Each spring, the Chinese labor market absorbs a graduating cohort larger than the one before it. The Ministry of Education projected 12.7 million new higher-education graduates for 2026, roughly 480,000 more than in the previous year, and the surveyed urban unemployment rate for 16- to 24-year-olds outside education stood at 17.9% in July 2026 (Xinhua News Agency, 2025; National Bureau of Statistics of China, 2026). Career research conducted in this setting has, understandably, framed the difficulty of choosing as a problem of scarcity: too few posts, too few employers willing to consider a given degree, too few routes that a family will accept. On that reading, the remedy is to widen the field of view, and career services are organized accordingly around information provision, employer fairs and the expansion of the option set a student is aware of.

The framing has a strong theoretical warrant. Social cognitive career theory treats contextual supports and barriers as proximal influences on the formation of choice goals and on the translation of goals into action (Lent et al., 1994, 2000), and its career self-management extension makes adaptive career behavior conditional on the resources a person can bring to bear (Lent & Brown, 2013; Carkit & Lent, 2026). Recent empirical evidence is consistent with this framework. Among female undergraduates at four non-elite Chinese universities, career anxiety was positively associated with career decision-making difficulties (CDD), and interviewees located that anxiety in an option set they experienced as shrinking (Pan & He, 2026). Resilience and general self-efficacy, both of which help a person act on the options available, are negatively associated with CDD among Chinese university students (Chang et al., 2026), and future anxiety, which is in part anxiety about a foreclosed opportunity structure, is positively associated with every CDD domain among emerging adults (Pajestka & Paszkowska-Rogacz, 2026).

Research on choice itself has spent twenty-five years documenting the opposite pattern. Offering people more alternatives can reduce the probability that they choose at all and lower their satisfaction with what they finally take (Iyengar & Lepper, 2000). The effect is not universal: a meta-analysis of 63 conditions put the mean effect of assortment size at approximately zero with substantial heterogeneity between studies (Scheibehenne et al., 2010), and a later synthesis of 99 observations resolved much of that heterogeneity by identifying four conditions under which large assortments become burdensome, namely a complex choice set, a difficult decision task, uncertainty about one’s own preferences, and the absence of a clear decision goal (Chernev et al., 2015). Both reviews have been extended recently (Jacob et al., 2024; Misuraca et al., 2024). What is striking is that a career decision satisfies all four conditions at once. Occupations differ on many incommensurable attributes, the decision is consequential and hard to reverse, young adults are by definition still learning what they want, and the goal is rarely more specific than a good job. Several features of contemporary labor markets are likely to strengthen these conditions. Platform and gig work fragment the routes into a career (Kost et al., 2020; Cropanzano et al., 2023), career shocks such as the COVID-19 pandemic can redirect careers in ways that are hard to anticipate (Akkermans et al., 2020), and generative artificial intelligence is expected to change the task content of a large share of occupations (Eloundou et al., 2024), which may leave young adults comparing options whose future is itself uncertain.

The two bodies of literature therefore make opposite predictions about the same variable over different parts of its range, which is a description of a non-monotone association. Career research has nonetheless almost always modeled the perceived breadth of the option set linearly, where it has modeled it at all, and in adjacent fields, curvature has frequently been tested by inspecting the sign and significance of a squared term, a procedure that is neither necessary nor sufficient for establishing a U shape (Lind & Mehlum, 2010; Haans et al., 2016). Establishing that both arms are present, and locating the point at which one gives way to the other, is the first task of this study.

A second task concerns mechanism. If a U shape exists, the two arms need not be produced by the same process, and a model that estimates only a squared term cannot tell them apart. Regret regulation theory supplies a candidate for the upward arm (Zeelenberg & Pieters, 2007). Regret is evaluated by comparing what one chose against what one might have chosen, so it depends on the forgone alternatives that remain attractive after the choice; because every additional live option must be weighed against each option already in view, the trade-offs a chooser must resolve multiply faster than the options themselves, so the regret a person anticipates should rise with the number of live options and may rise faster as that number grows. Career regret is not a trivial outcome: it forms around a realized loss that the person traces back to a specific earlier decision, and it reshapes subsequent career behavior (Schulze Schleithoff et al., 2026). If anticipated career regret carries the curvature, then a wider option set that is associated with less difficulty for an option-poor young adult would be expected to impose greater decisional costs on an option-rich one, and the model can make that expectation explicit rather than leaving it inside a squared term.

A third task concerns who turns earliest. The costs of a large option set are not borne equally. People who search alternatives exhaustively in pursuit of the best available outcome, rather than stopping at one that is good enough, report more regret and less satisfaction with what they obtain (Schwartz et al., 2002), and graduating seniors high on this maximizing tendency accepted jobs paying about 20% more while feeling worse about them throughout the search (Iyengar et al., 2006). If maximizing steepens the link from option breadth to anticipated regret, it should move the turning point of the whole curve toward a smaller option set, so that the location of the turning point would be a property of the chooser as much as of the choice.

This study examines these three questions in a three-wave time-lagged survey of Chinese young adults who were making a career decision, in which the predictor and moderators, the mediator and the outcome were measured on separate occasions (Section 3.1). It is designed to make three contributions. First, it tests whether the association between perceived option breadth and CDD is U-shaped, using a test appropriate to that hypothesis, and locates the turning point in the interpretable metric of occupational options under active consideration. Second, it asks whether the curvature can be decomposed into a direct component and a component carried by anticipated regret, which would identify a candidate mechanism for the ascending arm. Third, it examines whether the location of the turning point depends on the chooser, moving to a narrower option set among those who search alternatives most exhaustively.

## 2. Theoretical Background and Hypotheses

The argument draws on several theories whose roles differ. Social cognitive career theory, and in particular its career self-management model, is the organizing framework: it treats a young adult’s appraisal of the options open to them as one of the contextual and self-referent inputs from which career goals are formed and acted upon (Lent & Brown, 2013, 2020), and CDD are difficulties in that process. The framework is usually read as implying that more perceived opportunity is always better; the other theories specify where that implication should hold and where it should fail. Gottfredson’s (1981) theory of circumscription and compromise is a developmental complement that explains why a narrow set of acceptable options is costly, and so grounds the descending arm of the association. Research on choice overload, together with Simon’s (1955) account of bounded rationality and satisficing (Artinger et al., 2022), explains why the cost of comparing alternatives rises with their number, and so grounds the ascending arm at the level of the option set (Chernev et al., 2015). Regret regulation theory specifies the psychological mechanism through which that cost is expected to reach the chooser (Zeelenberg & Pieters, 2007), and the maximizing literature identifies who should be most exposed to it (Schwartz et al., 2002; Turner et al., 2012).

### 2.1. Career Decision-Making Difficulties

CDD are conceptualized here using the taxonomy of Gati et al. (1996), which organizes the obstacles a person meets when making a career decision into three clusters: a lack of readiness, comprising low motivation, general indecisiveness and dysfunctional beliefs; a lack of information about the decision process, the self, the occupations available and the ways of obtaining further information; and inconsistent information, comprising unreliable information together with internal and external conflicts. The structure implied by the taxonomy has been supported across 13 countries and nine language versions of the Career Decision-Making Difficulties Questionnaire (CDDQ; Levin et al., 2023), and the difficulties it measures are reliably associated with lower self-evaluations, most strongly with process-related self-efficacy (Udayar et al., 2020). They are also not confined to the undecided: among students who described themselves as career-decided, close to half reported discomfort with their choice and difficulty scores comparable to those of their undecided peers (Arbona et al., 2024).

What the taxonomy does not settle is where the difficulties come from. Within social cognitive career theory, the answer is a shortage of the personal and contextual resources needed to form and pursue a goal (Lent et al., 1994, 2000; Lent & Brown, 2013), and the empirical literature, including studies of Chinese samples, has largely followed that logic, relating CDD to anxiety, social support, parental involvement, personality and self-efficacy (Zhou et al., 2024; Aydemir Dev & Bayram Arlı, 2025; Chang et al., 2026; Pan & He, 2026). In that work, the size of the option set enters, if at all, as a resource: the more that is open to a person, the fewer the difficulties expected.

### 2.2. Option Breadth: Two Opposing Predictions and a U Shape

Perceived career option breadth is defined here as the range of occupational alternatives a young person regards as realistically open to them. It is not a new construct in kind. It is closest to Gottfredson’s (1981) zone of acceptable alternatives, the band of occupations a person regards as compatible with their self-concept and attainable, and its behavioral counterpart is the consideration set of decision research, which is the subset of alternatives a chooser actively evaluates (Hauser & Wernerfelt, 1990). What the present operationalization adds is a focus on perceived realistic accessibility across occupations. This distinguishes it from three neighboring constructs: career exploration, the activity of gathering information about oneself and the world of work (Stumpf et al., 1983); perceived career opportunities, the perceived availability of job and career opportunities within one’s current employing organization (Kraimer et al., 2011); and perceived employability, a person’s appraisal of their chance of obtaining a job in the internal or external labor market rather than the range of occupations they regard as open (Forrier et al., 2015). Two arguments bear on its association with CDD, and they point in opposite directions.

The first is the resource argument. A person who perceives a wider field has more scope for matching an occupation to their interests and abilities, is less exposed to the possibility that the single acceptable route closes, and can therefore form and revise goals with more confidence. Gottfredson’s (1981) account of circumscription and compromise makes the point developmentally: careers are chosen by progressively eliminating alternatives from a zone of acceptable ones, and a person whose zone has already been narrowed by circumstance has little left with which to compromise. The prediction is that difficulties fall as perceived breadth increases.

The second is the overload argument. Choosing requires comparing alternatives on attributes that do not reduce to a common scale, and the cognitive cost of doing so rises faster than the number of alternatives; beyond some point, a chooser satisfices, defers or avoids (Simon, 1955). Simon’s account was framed for a chooser with limited time and computational capacity facing more alternatives than can be evaluated, and recent work treats satisficing as a current account of adaptive choice under uncertainty rather than a historical one (Artinger et al., 2022); contemporary career decisions, made under uncertainty about both the self and the future of occupations (Gati & Kulcsár, 2021), fit that description closely. The empirical literature is clear that this does not happen everywhere (Scheibehenne et al., 2010), and it is equally clear about the conditions under which it does: complex sets, difficult tasks, uncertain preferences and unspecified goals (Chernev et al., 2015; Jacob et al., 2024; Misuraca et al., 2024). A large replication program has also shown that conflict among options does not reliably produce deferral on its own (Evangelidis et al., 2023), which is why the conditions matter. The prediction is that difficulties rise as perceived breadth increases past the point at which those conditions begin to bind.

Neither argument is a claim about the whole range. The resource argument is most plausible where the option set is small enough that its expansion changes what is feasible; the overload argument is most plausible where the set is already large enough that its expansion changes only what must be compared. Taken together, they imply that the association is negative at low levels of perceived breadth, positive at high levels, and passes through an interior minimum. Establishing that pattern requires more than a significant squared term: the slope must be shown to be negative at the low end of the observed range and positive at the high end, and the extremum must lie inside that range (Lind & Mehlum, 2010; Haans et al., 2016).

**H1.**  *Perceived career option breadth is associated with career decision-making difficulties in a U-shaped manner: the association is negative at low levels of breadth, positive at high levels, and the turning point lies within the observed range*.

### 2.3. Anticipated Career Regret as a Candidate Carrier of the Curvature

Regret is the emotion produced by comparing what one has with what one would have had under a different choice, and anticipated regret is the version of it that operates before the decision, shaping which alternatives a person is willing to commit to (Zeelenberg & Pieters, 2007). Its size depends on how the chosen option compares with the forgone ones and in particular on how many forgone alternatives remain attractive enough to invite the comparison. This has a direct implication for option breadth. A graduate choosing between two plausible occupations, say a teaching post and a civil-service post, forgoes one alternative whose attractions are easy to keep in view. A graduate weighing eight or ten similarly attractive options, from product management and data analysis to postgraduate study abroad, forgoes seven or nine, and at least one of them is likely to look better than the chosen option on some attribute that matters. Anticipated regret should therefore increase with perceived option breadth. It may also increase at an accelerating rate. Every additional live option must be weighed against each option already in view, so the trade-offs a chooser must resolve before any option can be confidently preferred grow faster than the number of options, and each unresolved trade-off leaves a forgone alternative that could turn out to have been better. Whether the increase is in fact convex is an empirical question, which H2 tests.

The second half of the mechanism is the more familiar one. A young adult who expects to regret whatever they choose has an incentive to postpone choosing, to keep alternatives alive, and to treat a provisional commitment as revocable, all of which are recorded on the CDDQ as difficulty rather than as strategy. Qualitative work on how career decisions turn into career regret is consistent with the sequence: regret is organized around a loss the person traces back to a decision (Schulze Schleithoff et al., 2026), which suggests that anticipating such a loss could make committing feel costly. Because the mediator is a nonlinear function of the predictor, the indirect association is not a single number; it is the instantaneous indirect effect, the product of the slope of the mediator at a given value of the predictor and the slope of the outcome on the mediator (Hayes & Preacher, 2010).

**H2.**  *Perceived career option breadth is positively and convexly associated with anticipated career regret, so that the association grows steeper as breadth increases*.

**H3.**  *Anticipated career regret is positively associated with career decision-making difficulties, and the instantaneous indirect association of option breadth with difficulties through regret increases with option breadth, while the direct association remains negative*.

### 2.4. Maximizing Tendency as a First-Stage Condition

Choosers differ in how much of the option set they insist on examining. Maximizers search alternatives extensively in pursuit of the best available outcome; satisficers stop at the first alternative that clears their standard (Simon, 1955; Schwartz et al., 2002). The distinction has been refined since it was introduced, and current practice separates the goal of obtaining the best outcome from the strategy of exhaustive alternative search, which is the component that carries most of the association with regret and dissatisfaction (Turner et al., 2012; Cheek & Schwartz, 2016). A recent meta-analysis confirms that maximizing is reliably associated with lower wellbeing (Belli et al., 2022), and when decision information is abundant, maximizers find it harder both to choose options and to reject them, so the set under consideration is not narrowed (Khare et al., 2021). It is the search component that matters here. A young adult who compares every live option against every other is, by construction, more exposed to attractive forgone alternatives and should therefore convert a given widening of the option set into a larger increment of anticipated regret.

The consequence is not only a steeper first stage. If regret carries the upward arm of the curve, then steepening the first stage moves the point at which the upward arm overtakes the downward one. The turning point is a ratio of the linear and quadratic components of the total association, and a first-stage moderator enters both; a maximizer would therefore be expected to reach the turning point while perceiving fewer options.

**H4.**  *Maximizing tendency moderates the association between perceived career option breadth and anticipated career regret, which is stronger at higher levels of maximizing; consequently, the turning point of the total association lies at a lower level of option breadth among stronger maximizers*.

### 2.5. Career Decision-Making Self-Efficacy as a Second-Stage Condition

Career decision-making self-efficacy (CDSE) is confidence in one’s capacity to complete the tasks a career decision requires: appraising oneself accurately, gathering occupational information, selecting a goal, planning, and solving problems (Taylor & Betz, 1983; Betz et al., 1996). It is the self-evaluation most consistently associated with lower career indecision, and process-related efficacy predicts indecision more strongly than generalized efficacy does (Udayar et al., 2020). Its relevance here is to the second stage rather than the first. Anticipated regret becomes decisional difficulty when a person doubts their capacity to handle the consequences of a choice that turns out badly; a young adult confident in the decision process has procedural grounds for treating an imperfect choice as correctable and should convert a given level of anticipated regret into less difficulty.

**H5.**  *Career decision-making self-efficacy moderates the association between anticipated career regret and career decision-making difficulties, which is weaker at higher levels of self-efficacy*.

### 2.6. The Present Study

**Figure 1:**
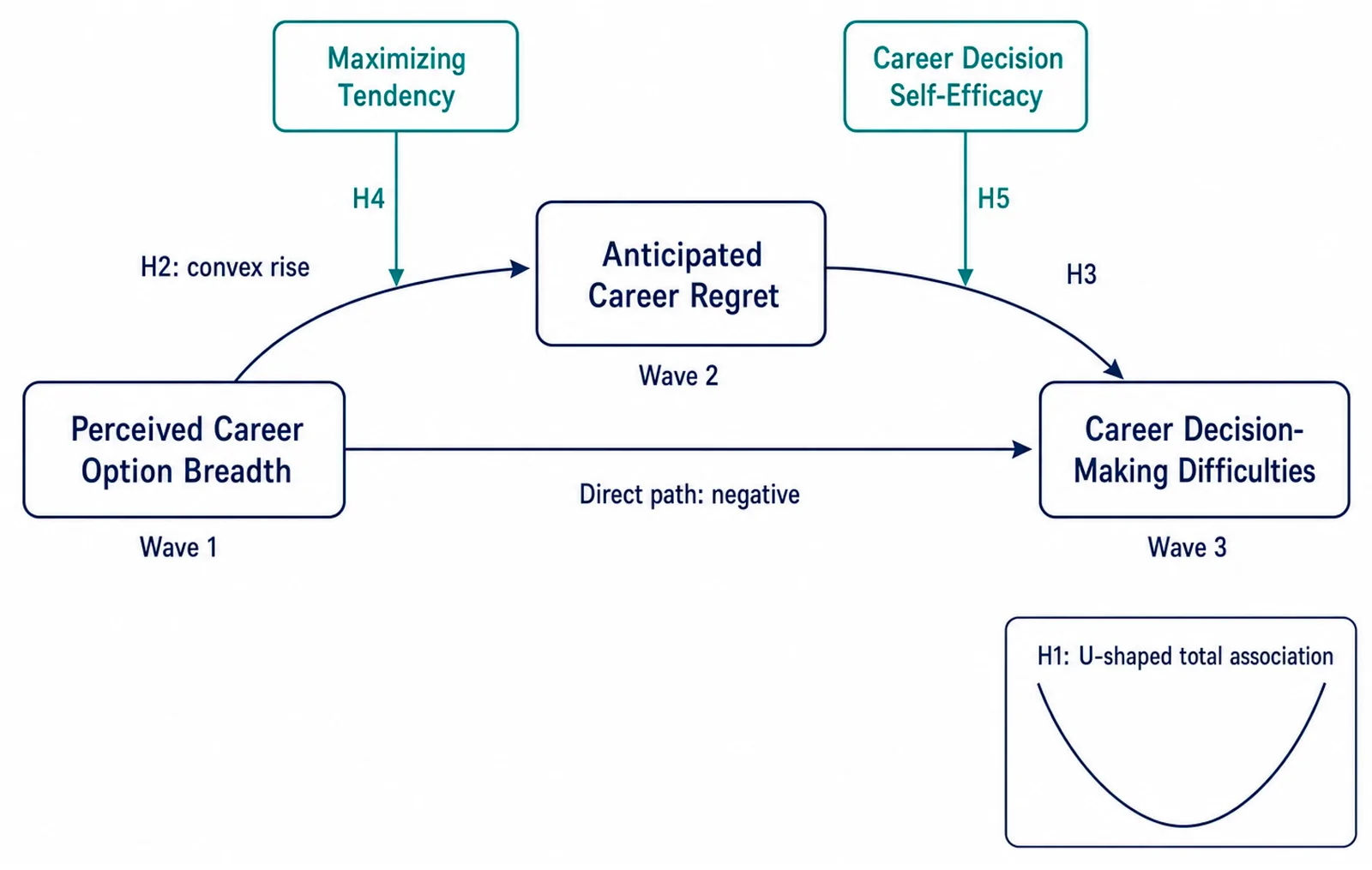
The hypothesized conditional process model. Note. Wave 1, Wave 2 and Wave 3 denote measurement occasions separated by four weeks. H2 states that the association of perceived career option breadth with anticipated career regret is positive and convex; H3 states that anticipated regret is positively associated with career decision-making difficulties, while the direct path remains negative. H4 and H5 are first-stage and second-stage moderation, respectively. H1 concerns the shape of the total association, which is shown in the inset. Covariates are omitted from the figure for clarity. Navy boxes and arrows denote the focal variables and the paths among them; teal boxes denote the two moderators, and teal arrows point to the paths they are hypothesized to moderate.

The five hypotheses form the conditional process model shown in Figure 1. Two features of the design deserve mention before the model is estimated. First, the predictor, the mediator and the outcome were measured on separate occasions (Section 3.1); this is a partial remedy for common method variance rather than a solution to it (Podsakoff et al., 2003, 2024). Second, the curvature is treated as the object of inference rather than as a nuisance: the U shape is tested with the bounds appropriate to it, the turning point is reported with a confidence interval and in an interpretable metric, and the indirect association is allowed to vary with the level of the predictor.

## 3. Materials and Methods

### 3.1. Design and Procedure

Data were collected in a three-wave time-lagged online survey between March and May 2026. The three questionnaires were separated by four weeks: Wave 1 ran from 9 to 23 March, Wave 2 from 6 to 20 April and Wave 3 from 4 to 18 May 2026. Perceived career option breadth, maximizing tendency, career decision-making self-efficacy, career exploration and the demographic covariates were measured at Wave 1; anticipated career regret at Wave 2; career decision-making difficulties at Wave 3. The interval was chosen to be long enough to break the retrieval context that produces consistency artifacts and short enough that the participants would still be in the same decision episode (Podsakoff et al., 2024). Three waves were used because the model specifies a three-step sequence. An appraisal of the option set (Wave 1) is expected to shape the regret a person anticipates when contemplating commitment (Wave 2), and that anticipation is expected to be expressed as difficulty in reaching a decision (Wave 3). Measuring each construct at the stage the model assigns to it builds the hypothesized temporal order into the design, which a two-wave design could do for only one of the two paths; it does not by itself establish causation or model change within persons (Section 6).

Participants were recruited through a certified academic survey panel and through the career-service mailing lists of cooperating universities in six cities chosen to span China’s principal labor-market regions: Hangzhou and Jiaxing in the Yangtze River Delta, Guangzhou in the Pearl River Delta, Tianjin in the Bohai Rim, Wuhan in central China and Chengdu in the west. Hangzhou, Guangzhou, Tianjin, Wuhan and Chengdu are provincial capitals or municipalities that serve as the principal graduate labor markets of their regions, and Jiaxing, where the authors’ institution is located, is a prefecture-level city, so the sample is not confined to the largest labor markets (149 to 216 participants per city in the analytic sample; Table 1). Both channels were used in all six cities: the academic panel invited eligible members resident in each city, and the career-service offices of cooperating universities in each city circulated the invitation. The analytic data set does not identify the channel through which each participant was recruited, so the effect of the channel could not be estimated directly; the study variables did not differ significantly across cities (all *p* ≥ 0.078, η^2^ ≤ 0.010), and city fixed effects left the results unchanged, although the curvature itself varied across cities (Section 4.6). Three eligibility criteria were applied at screening: an age between 18 and 35; enrollment in the final year of an undergraduate or master’s program, or employment of no more than three years’ standing; and an affirmative answer to the item “Are you currently making a decision about your career direction?”. The upper age limit corresponds to the definition of youth in China’s national youth development plan, which covers ages 14 to 35 (Central Committee of the Communist Party of China & State Council of the People’s Republic of China, 2017), the lower limit restricts the sample to adults, and the status criteria restrict the sample to people who are about to make, or have recently made, the transition from education to work, which is the period in which the choice of an occupational direction is most immediate; the limit of three years’ employment keeps early-career workers close to that transition. Participants were told that the study concerned how young adults think about career choices, that participation was voluntary and could be discontinued at any point, and that responses would be reported only in aggregate. Electronic informed consent was obtained before the Wave 1 questionnaire opened, and a small payment was made after each completed wave.

Of 1732 invitations issued at Wave 1, 1412 questionnaires were returned. Responses were discarded if the questionnaire was completed in under 180 s, if either of two embedded attention checks was failed, or if a participant gave the same response to every item of a multi-item scale, leaving 1268 valid Wave 1 records. Of these, 1127 completed Wave 2 and 1036 completed all three waves, which is a retention rate of 81.7% from Wave 1. The 232 respondents who supplied Wave 1 data but did not complete the study were compared with the analytic sample on eleven Wave 1 variables. None of the eleven comparisons reached significance, the smallest *p* being 0.075 (age), so attrition is treated as unrelated to the observed characteristics. Because the online instrument required a response to every item, there were no item-level missing data within a completed wave, and all analyses use the complete three-wave sample.

The study was conducted in accordance with the Declaration of Helsinki, and the protocol was approved in advance by the Academic Ethics Committee of Jiaxing University. An a priori power analysis was conducted in G*Power 3.1 (linear multiple regression, fixed model, *R*^2^ increase) for the test of a single added term in a model with 16 predictors, which was at least as many as any of the hypothesis-testing equations (13 in the reduced form, 15 in the mediator and outcome models). It indicated that 395 cases were needed to detect an increment of *f*^2^ = 0.02, the conventional small effect (Cohen, 1988), with 80% power at α = 0.05. Because moderating effects of categorical variables in published regression research have typically been far smaller than this benchmark (Aguinis et al., 2005) and interactions between continuous variables are difficult to detect in non-experimental data (McClelland & Judd, 1993), sensitivity analyses were also run for the tests actually conducted. With *N* = 1036, the smallest increments detectable with 80% power were *f*^2^ = 0.0076 for single-term tests and 0.0093 for the two-term test of H4, and the simulated power for effects of the size observed was 0.98 for the quadratic term of H1, 0.86 for H4 and 0.51 for H5 (Appendix A, Table A2).

### 3.2. Participants

Table 1 describes the analytic sample. Although eligibility extended to age 35, the realized sample was concentrated in the early-to-mid twenties (*M* = 23.69, *SD* = 3.01, IQR 22–26, range 18–33; 98.7% aged 30 or younger). Slightly more than half of the participants were women, and just under two thirds were still studying, the remainder being in employment of no more than three years’ standing. More than half held urban household registration and more than half were the first in their family to enter university. The median participant was weighing 5 distinct occupations at Wave 1 (IQR 3–6), which is a quantity that becomes the interpretable metric for the turning point reported in Section 4.2.

### 3.3. Measures

All instruments not originally written in Chinese were prepared following the standard procedure for the cross-cultural adaptation of self-report measures (Beaton et al., 2000). Two bilingual postgraduates in psychology translated each instrument independently and reconciled their versions; two further bilingual researchers, blind to the source, back-translated the reconciled version; an expert panel comprising the translators, a psychometrician and two career counselors reviewed semantic, idiomatic and conceptual equivalence; and cognitive interviews using a think-aloud protocol were conducted with 18 young adults drawn from the target population before the wording was fixed. The CDDQ was administered in its published Chinese revision (Wu et al., 2016) and did not require translation. Two outcomes of this process are noted with the relevant measures below: Maximization Inventory items with consumer-context examples were adapted to a career-relevant wording, and the wording of the option-breadth items written for this study was refined in the cognitive interviews. No other items were changed, the expert panel did not identify conceptual difficulties, and no further cultural adaptation was required. Each instrument is described below with its source, its internal consistency and, for the two scales written for this study, its factor structure; all other construct-validity evidence from the present sample is reported together in Section 4.1 and Appendix A. Prior validation evidence for the established instruments comes from their development studies (Stumpf et al., 1983; Betz et al., 1996; Turner et al., 2012) and, for the CDDQ, from its Chinese revision (Wu et al., 2016) and a 13-country test of its structure (Levin et al., 2023). Because the option-breadth and anticipated-regret scales were written for this study and have not been validated in an independent sample, results that depend on them should be treated as provisional (Section 6).

*Perceived career option breadth (Wave 1).* Existing operationalizations of the zone of acceptable alternatives and of consideration sets record which occupations are acceptable or under consideration rather than how many kinds of work are perceived as realistically open, and no instrument measures the perceived size of a young adult’s live option set; breadth was therefore assessed in two ways. The first was a five-item scale written for this study from the definition given in Section 2.2 and refined in the cognitive interviews, which was answered on a seven-point scale from 1 (strongly disagree) to 7 (strongly agree). Before administration, 6 experts rated the relevance of each item to the definition on a four-point scale; item-level content validity indices ranged from 0.83 to 1.00, and the scale-level index was 0.97. Sample items are “There are many different kinds of work that I could realistically move into” and “If my current plan does not work out, several other lines of work are genuinely open to me”. The second was a single count item, “How many distinct occupations or job types are you seriously considering at the moment?”, which was answered as an integer. The scale is used as the primary measure and the count as a parallel measure that expresses the turning point in an interpretable metric; the two correlate at *r* = 0.71. They share about half their variance and are not interchangeable: the scale captures a general sense of how many kinds of work are realistically open, whereas the count records the options actively being weighed, so the 6.5-option turning point, obtained from a parallel model in which the count replaced the scale, is a related estimate in an interpretable unit rather than a replication of the scale result. For the scale, Cronbach’s α = 0.873, McDonald’s ω = 0.874, and a one-factor confirmatory model gave CFI = 0.976, TLI = 0.952 and SRMR = 0.027 with standardized loadings between 0.69 and 0.80. RMSEA was 0.106 (χ^2^(5) = 63.43), but the misfit was confined to a single residual correlation between items 2 and 4 (0.06); with that residual covariance freed, the model fitted closely (χ^2^(4) = 1.77, CFI = 1.000, RMSEA = 0.000). This covariance was not specified in advance, and modifications suggested by the data can capitalize on chance (MacCallum et al., 1992). Within this sample, it was consistent, although random halves are not independent replications: it appeared in both halves of the random split used in Section 4.1 (0.06 in each) and in every half of 1000 further random splits, and removing either item removed the misfit (RMSEA = 0.000), whereas removing any other item did not (Appendix A, Table A1). Because the misfit is confined to this pair and parallel analysis in a random half retained a single factor, we interpret the covariance as a minor source of specific variance that items 2 and 4 share beyond the common factor (correlation between their unique factors 0.31), not as a dimension that would call for separate scoring; fit statistics cannot distinguish a wording effect from a narrow content facet, and whether the covariance recurs in new samples remains to be examined (Section 6). With either or both items removed, or with factor scores in place of the item mean, the conclusions for H1 to H4 were unchanged, whereas the product term for H5, just short of significance in the reported model (*p* = 0.055), ranged from *p* = 0.042 to 0.059 (Section 4.5).

*Anticipated career regret (Wave 2).* Six items written for this study from the regret-regulation account of anticipated regret (Zeelenberg & Pieters, 2007) asked participants how much they expected to regret the career decision they were currently facing, and were answered from 1 (not at all) to 7 (very much). Sample items are “I expect that I will keep wondering whether another line of work would have suited me better” and “Whatever I choose, I think I will worry that I passed up something better”. Cronbach’s α = 0.885, ω = 0.886. A one-factor confirmatory model gave CFI = 0.984, TLI = 0.974, RMSEA = 0.071 and SRMR = 0.023 with standardized loadings between 0.70 and 0.81. Because some items refer to continuing worry about a better alternative, the scale’s distinctness from CDD, which include indecision and internal conflict, was examined directly (Section 4.1).

*Maximizing tendency (Wave 1).* The alternative-search component of maximizing was measured with seven items from the Maximization Inventory (Turner et al., 2012); this is the component that current measurement work identifies as carrying the association with regret rather than the high-standards component (Cheek & Schwartz, 2016). Items were answered from 1 (strongly disagree) to 5 (strongly agree); a sample item, shown in its original wording, is “I take the time to read the whole menu when dining out”; in the administered version, its dining context was replaced by a career-choice context following the cognitive interviews. Cronbach’s α = 0.877, ω = 0.877.

*Career decision-making self-efficacy (Wave 1).* The 25-item short form of the Career Decision Self-Efficacy Scale (Betz et al., 1996) covers the five task domains of accurate self-appraisal, gathering occupational information, goal selection, planning and problem solving; items were answered from 1 (no confidence at all) to 5 (complete confidence). Cronbach’s α = 0.956, ω = 0.956.

*Career decision-making difficulties (Wave 3).* The Chinese revision of the CDDQ (Wu et al., 2016), based on the taxonomy of Gati et al. (1996), comprises 36 items across the three clusters of lack of readiness, lack of information and inconsistent information, answered from 1 (strongly disagree) to 5 (strongly agree), with higher scores indicating greater difficulty. Cronbach’s α = 0.961, ω = 0.961; the three cluster scores are analyzed separately in Section 4.6.

*Covariates.* Career exploration was measured with six items from the Career Exploration Survey (Stumpf et al., 1983) and is included because a young adult who has searched more widely both perceives more options and is further into the decision process; without it, the association of interest would be confounded with search behavior. Cronbach’s α = 0.861. Age, gender (recorded as woman or man), current status (student or early-career worker), highest qualification, household registration, first-generation university status, family income and only-child status were also recorded at Wave 1 and are included in every model.

### 3.4. Common Method Variance

Three procedural remedies were used. The predictor, the mediator and the outcome were measured on separate occasions four weeks apart; response formats were deliberately varied across constructs, with seven-point scales for option breadth (agreement) and anticipated regret (extent) and five-point scales for the remaining measures; and anonymity was guaranteed at each wave (Podsakoff et al., 2003).

Two statistical diagnostics were then run with the caveats that current reviews attach to both (Podsakoff et al., 2024). Harman’s single-factor test placed 23.34% of the variance in the first unrotated component, below the conventional 40% threshold, although this test is insensitive and is reported only as a screen. More informatively, a single-factor confirmatory model on which every indicator loaded fitted the data very poorly (χ^2^/*df* = 30.29, CFI = 0.422, TLI = 0.382, RMSEA = 0.168), whereas the five-factor measurement model reported in Section 4.1 fitted well. Method variance is nonetheless certain to be present, and the temporal separation of the waves reduces rather than removes it.

### 3.5. Data Analysis

The analyses proceed in six steps; the first five test H1 to H5, and the sixth examines the robustness of the turning point. (1) The reduced form, Equation (3), tests whether perceived option breadth has a U-shaped association with CDD (H1), using the bounds test of Equation (7) and the turning point of Equation (5). (2) The mediator model, Equation (1), tests whether option breadth is positively and convexly associated with anticipated regret (H2). (3) The outcome model, Equation (2), with the instantaneous indirect effect of Equation (6), tests whether anticipated regret is associated with CDD and carries part of the curvature, while the direct association remains negative (H3). (4) The product terms of Equation (1) test whether maximizing tendency moderates the first stage and, through it, the location of the turning point (H4). (5) The product term of Equation (2) tests whether CDSE moderates the second stage (H5). (6) The location of the turning point and its sensitivity to specification, influential observations, range restriction, and subgroup and city differences are then examined (Section 4.6). Preliminary analyses (attrition, descriptive statistics and common method variance) and the measurement model precede these steps (Section 3.1, Section 3.4 and Section 4.1). Confirmatory factor models were estimated by maximum likelihood.

All variables entering products were standardized before the products were formed, which removes the non-essential collinearity that otherwise makes the lower-order terms uninterpretable and expresses the coefficients in standard-deviation units. The largest variance inflation factor in the full outcome model was 1.28. Coefficients are reported with heteroskedasticity-consistent (HC3) standard errors. Two regressions define the conditional process model. The mediator model is

(1) $$R_{i}=\alpha_{0}+\alpha_{1}B_{i}+\alpha_{2}B_{i}^{2}+\alpha_{3}M_{i}+\alpha_{4}B_{i}M_{i}+\alpha_{5}B_{i}^{2}M_{i}+c’X_{i}+\varepsilon_{i}$$

where *R* is anticipated career regret at Wave 2, *B* is perceived career option breadth at Wave 1, *M* is maximizing tendency at Wave 1, and ***X*** collects career decision-making self-efficacy, career exploration and the demographic covariates (bold type denotes vectors; ***c***, ***d*** and ***f*** in Equations (1)–(3) are the corresponding coefficient vectors, and the prime denotes the transpose). The outcome model is

(2) $$D_{i}=\gamma_{0}+\gamma_{1}B_{i}+\gamma_{2}B_{i}^{2}+\gamma_{3}R_{i}+\gamma_{4}E_{i}+\gamma_{5}R_{i}E_{i}+d’X_{i}+\nu_{i}$$

where *D* is career decision-making difficulties at Wave 3 and *E* is career decision-making self-efficacy; in Equations (2) and (3), ***X*** also contains maximizing tendency, and in Equation (2), self-efficacy enters through *E* rather than through ***X***. The total association of option breadth with difficulties, which is what H1 concerns, is estimated from the reduced form

(3) $$D_{i}=\beta_{0}+\beta_{1}B_{i}+\beta_{2}B_{i}^{2}+f’X_{i}+u_{i}$$

in which the mediator is deliberately omitted, so that the marginal association

(4) $$\frac{\partial E\left[D_{i}|B_{i}\right]}{\partial B_{i}}=\beta_{1}+2\beta_{2}B_{i}$$

carries both components. The extremum of the fitted curve is

(5) $$B^{*}=\frac{-\beta_{1}}{2\beta_{2}}$$

for which a delta-method standard error and a Fieller interval are reported, the latter because the turning point is a ratio of estimated coefficients and the delta method is known to understate its uncertainty. Because option breadth enters Equation (1) quadratically, the indirect association is not constant; following Hayes and Preacher (2010), it is summarized by the instantaneous indirect effect

(6) $$\theta \left(B,M,E\right)=\left(\alpha_{1}+2\alpha_{2}B+\alpha_{4}M+2\alpha_{5}BM\right)\left(\gamma_{3}+\gamma_{5}E\right)$$

whose percentile 95% confidence intervals are obtained from 5000 nonparametric bootstrap resamples, the resampling being preferred to a normal-theory test because the product of two coefficients has a skewed sampling distribution (Hayes, 2018). Finally, because a significant quadratic coefficient is neither necessary nor sufficient for a U shape over an observed interval, H1 is tested against the composite null

(7) $$H_{0}:\left(\beta_{1}+2\beta_{2}B_{min}\geq 0\right)\cup \left(\beta_{1}+2\beta_{2}B_{max}\leq 0\right)$$

which is rejected only when the fitted slope is significantly negative at the lower bound of the observed data and significantly positive at the upper bound (Lind & Mehlum, 2010; Haans et al., 2016). The reported statistic is the less favorable of the two one-sided tests. Analyses were carried out in Python 3.13 using statsmodels and semopy; the random seed for every resampling procedure is fixed in the analysis script.

## 4. Results

### 4.1. Measurement Model, Reliability and Descriptive Statistics

Table 2 reports the descriptive statistics and internal consistency coefficients of the study variables. The five-factor measurement model, estimated with the items of the option-breadth, maximizing and regret scales and with parcels formed from the subscales of the self-efficacy scale and the clusters of the CDDQ, fitted the data well (χ^2^ = 667.92, *df* = 454, χ^2^/*df* = 1.47, CFI = 0.991, TLI = 0.990, RMSEA = 0.021, GFI = 0.972). Composite reliability exceeded 0.87 for every construct, and the average variance extracted exceeded 0.50 for the three scales entered at item level (Table 3). For the two long instruments, the item-level average variance extracted fell below that threshold (0.46 for self-efficacy and 0.41 for career decision-making difficulties), which is common in instruments of 25 and 36 items covering several content domains; convergent validity is nonetheless supported because composite reliability is well above 0.60 in both cases (Fornell & Larcker, 1981), and at the parcel level on which the model was estimated, the average variance extracted was 0.82 and 0.73, respectively. The parcels follow the instruments’ content: the five CDSE parcels are its task-domain subscales (items 1–5, 6–10, 11–15, 16–20 and 21–25), and the nine CDDQ parcels are means of four consecutive items, three within each cluster (items 1–12, 13–24 and 25–36). Modeling the parcels of each instrument as indicators of one factor presupposes that a single common factor accounts for their covariation, and the use of total scores in the hypotheses does not by itself establish this, so the decision to do so rests on the evidence. The domains and clusters were not empirically separable in this sample (cluster scores correlated 0.89 to 0.90, domain scores 0.81 to 0.84, and latent correlations reached or slightly exceeded 1.00 when they were modeled as separate factors). Because parcels can mask item-level misfit (Little et al., 2013; Marsh et al., 2013), the model was re-estimated with all 79 items as indicators (CFI = 0.969, RMSEA = 0.020, SRMR = 0.026; loadings 0.49 to 0.81) and with interleaved parcels (CFI = 0.971, RMSEA = 0.036); fit remained acceptable (Appendix A, Table A1). The item-level model, in which every item loads directly on the single factor of its instrument, provides the most direct support in these data for treating each instrument as essentially unidimensional, and it did not fit significantly worse than the model that separated the domains and clusters (Δχ^2^(45) = 58.04, *p* = 0.092), whose solution was improper (latent correlations of 1.00 or more). Discriminant validity was supported on both criteria (Table 3): the square root of the average variance extracted exceeded every corresponding correlation, and the largest heterotrait–monotrait (HTMT) ratio among the five constructs of the measurement model was 0.31, which is far below the 0.85 benchmark (Henseler et al., 2015). Further checks concern the two scales written for this study (Appendix A, Table A1). In one random half of the sample, exploratory factor analysis of the option-breadth, career-exploration and anticipated-regret items retained three factors, with primary loadings of 0.66 or more and a largest cross-loading of 0.07, and the three-factor model was confirmed in the other half (CFI = 0.981, RMSEA = 0.036). Option breadth was distinct from career exploration (latent *r* = 0.33; merging the factors worsened fit, Δχ^2^(1) = 1982.37, *p* < 0.001), anticipated regret was distinct from CDD (latent *r* = 0.30; Δχ^2^(1) = 2664.72, *p* < 0.001; HTMT 0.29 to 0.31 with each cluster), and, as expected, anticipated regret correlated positively with maximizing tendency (latent *r* = 0.28). These checks use the same sample as the hypothesis tests and do not substitute for independent validation (Section 6).

Table 2 also gives the zero-order correlations. Perceived option breadth was negatively correlated with career decision-making difficulties (*r* = −0.177, *p* < 0.001), which is the linear pattern the resource argument predicts, and positively correlated with anticipated career regret (*r* = 0.275, *p* < 0.001), which is not. Anticipated regret was positively correlated with difficulties (*r* = 0.278, *p* < 0.001) and self-efficacy negatively so (*r* = −0.299, *p* < 0.001). Univariate distributions were acceptably normal with all absolute skewness below 1 and all absolute kurtosis below 1 (Kline, 2023).

### 4.2. The Shape of the Association Between Option Breadth and Difficulties

Table 4 builds the reduced form in four steps. Column (1) contains the covariates alone. Column (2) adds perceived option breadth linearly; its coefficient is negative and significant (*b* = −0.171, *SE* = 0.034, *p* < 0.001), which is the result a study fitting a linear model would report. Column (3) adds the squared term. It is positive and significant (*b* = 0.097, *SE* = 0.026, *p* < 0.001) and adds 1.7% to the explained variance, while the linear term remains negative. Column (4) adds maximizing tendency, self-efficacy and career exploration; the quadratic term is essentially unchanged (*b* = 0.089, *SE* = 0.024, *p* < 0.001, unique Δ*R*^2^ = 0.014), and the linear term remains negative (*b* = −0.117, *SE* = 0.032, *p* < 0.001). Column (4) is the reduced form used throughout.

A positive squared coefficient is not by itself evidence of a U shape, since the extremum may lie outside the observed data, or one arm may be flat (Haans et al., 2016). Table 5 therefore reports the test of Equation (7) together with the location of the turning point. At the lowest observed value of option breadth, the fitted slope was −0.648 (*SE* = 0.146, *t* = −4.44, *p* < 0.001); at the highest, it was 0.315 (*SE* = 0.119, *t* = 2.65, *p* = 0.004). The composite null of Equation (7) is therefore rejected (*t* = 2.65, *p* = 0.004), and H1 is supported. The evidence for the two arms is not symmetric, however. The slope was significantly negative over the lower 60.7% of the observed distribution of option breadth but significantly positive only above 1.52 standard deviations, a region containing 6.0% of participants (*n* = 62), which is also where the upper bound of the test lies. The turning point lies at 0.66 standard deviations above the mean of option breadth, with a Fieller 95% interval of [0.28, 1.52] and a percentile bootstrap interval of [0.28, 1.56], both of which lie inside the observed range of −2.99 to 2.43. Just under three quarters of the sample (72.9%) lay below the estimated turning point and 27.1% above it.

**Figure 2:**
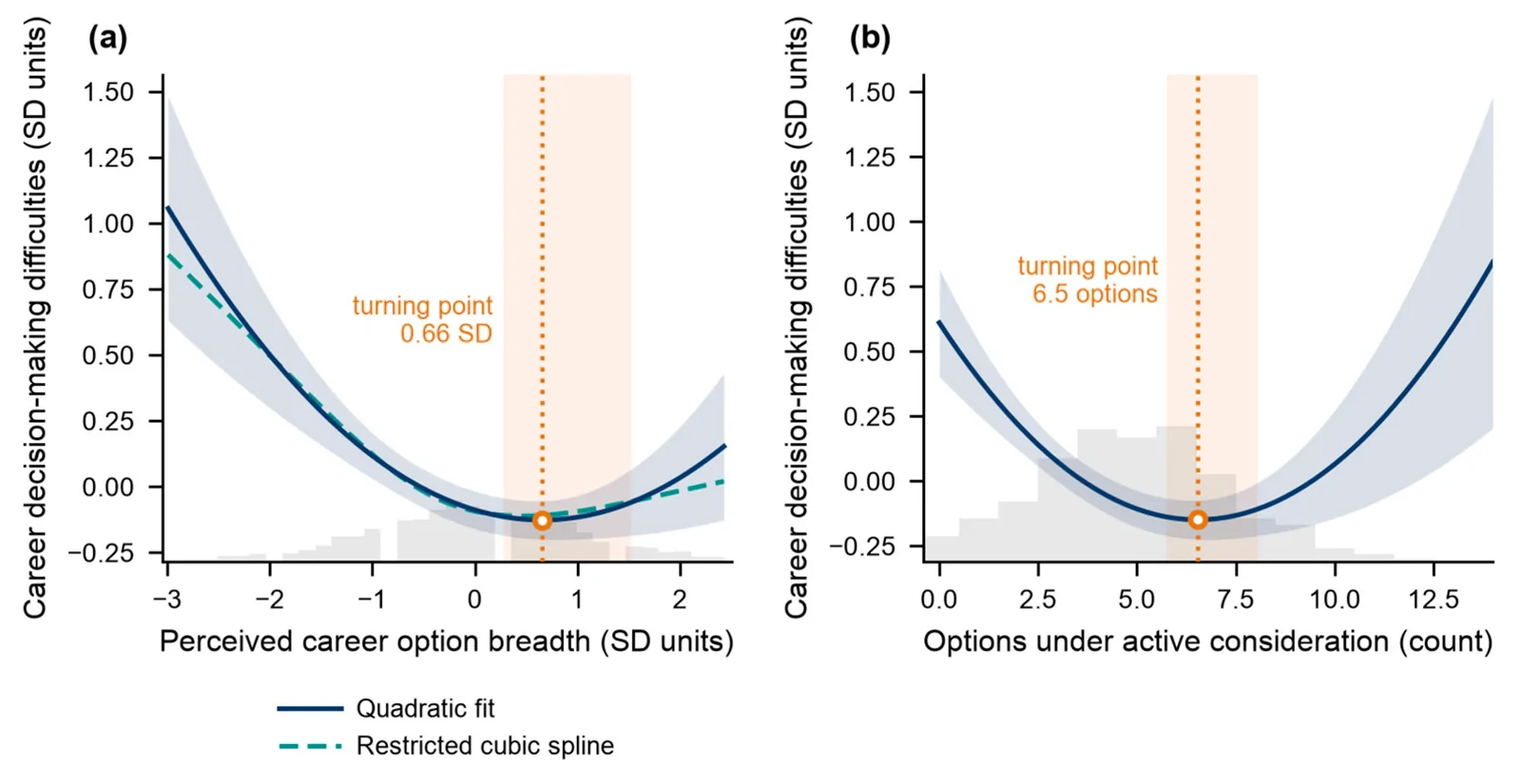
Fitted association between perceived career option breadth and career decision-making difficulties. (**a**) Standardized scale metric with the restricted cubic spline shown for comparison; (**b**) the metric of occupational options under active consideration. Note. Shaded bands are 95% confidence intervals for the fitted values; the vertical dotted line marks the turning point and the pale vertical band its 95% Fieller interval; the gray histogram shows the distribution of option breadth. Estimates come from column (4) of Table 5 (panel a) and from the corresponding count-metric specification (panel b), both with covariates held at their means. *N* = 1036.

Figure 2 plots the fitted curve. Panel (a) uses the standardized scale metric and overlays a restricted cubic spline with four knots, which is not constrained to be quadratic and traces almost the same path. Panel (b) repeats the exercise in the metric of the participant’s own count of occupations under active consideration, which is the form in which the result is easiest to state: the fitted minimum lay at 6.5 options (95% Fieller interval [5.75, 8.05]), and 23.5% of participants were weighing more than that. This value locates the minimum of a fitted curve; it is not a threshold separating better-off from worse-off groups, and participants weighing seven or more options did not report more difficulties than those weighing six or fewer (unadjusted, they reported significantly fewer, *d* = −0.19, and the adjusted difference was not significant; Appendix A, Table A3). The median participant was considering 5 options (IQR 3–6).

**Figure 3:**
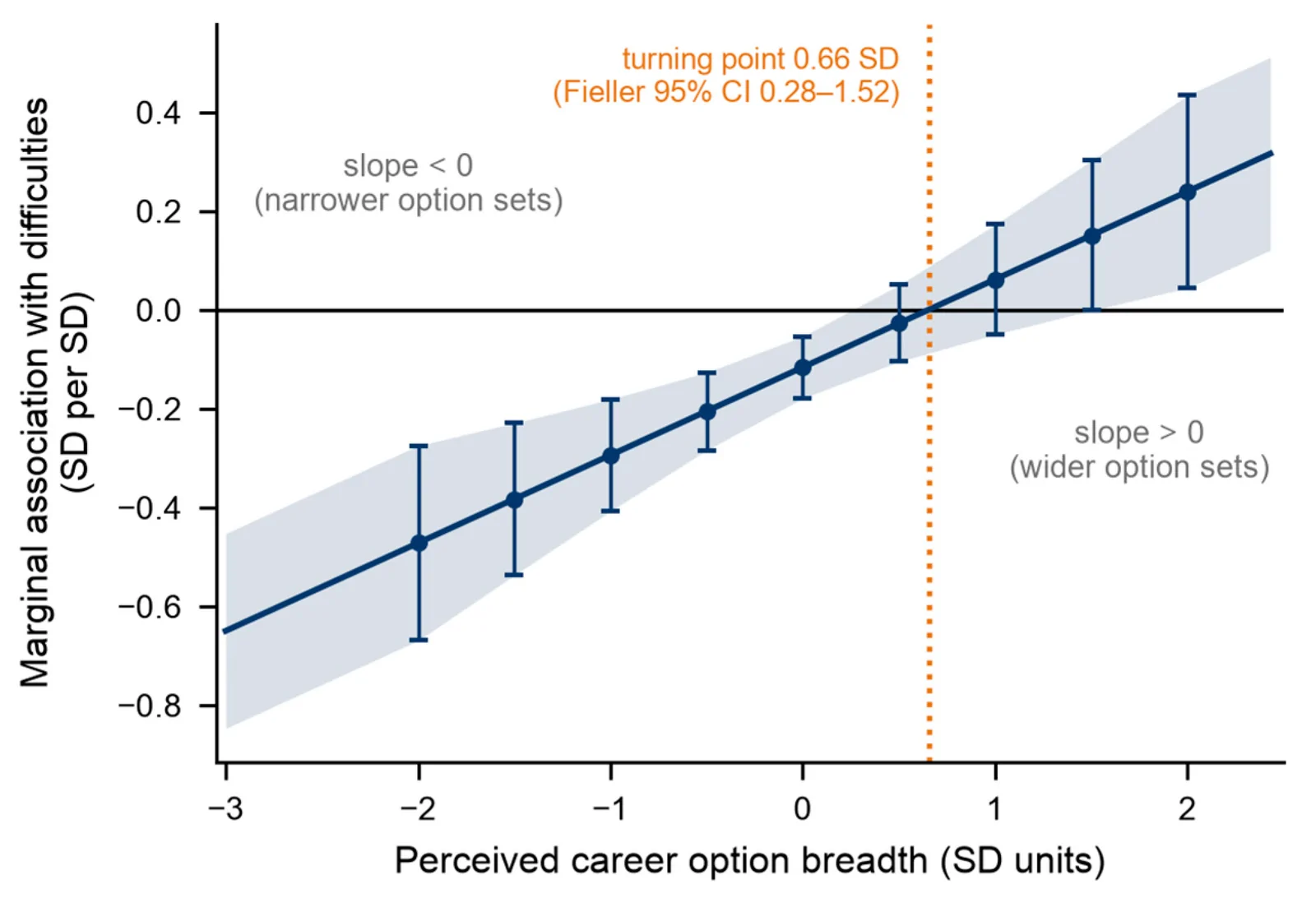
Marginal association of perceived career option breadth with career decision-making difficulties across the observed range. Note. The solid line is the marginal association of Equation (4) and the shaded band its 95% confidence interval; points with bars are the same quantity evaluated at half-standard-deviation intervals. The vertical dotted line marks the turning point, and the annotation gives its Fieller 95% interval. Estimates are from column (4) of Table 4. *N* = 1036.

Figure 3 shows the same result as the marginal association of Equation (4). The slope was −0.349 at the tenth percentile of option breadth (*p* < 0.001), −0.250 at the twenty-fifth (*p* < 0.001) and −0.117 at the median (*p* < 0.001); it was indistinguishable from zero at the seventy-fifth percentile (0.016, *p* = 0.734) and positive but not significant at the ninetieth (0.116, *p* = 0.095). The pattern is asymmetric. In the bottom quarter of the option-breadth distribution, a one-standard-deviation difference in perceived breadth was associated with at least one quarter of a standard deviation less difficulty (−0.25 at the 25th percentile and −0.35 at the 10th); near the seventy-fifth percentile, the association was close to zero, and it was significantly positive only in the upper tail (above 1.52 standard deviations).

### 4.3. Anticipated Career Regret and the Instantaneous Indirect Association

Table 6 reports the two equations of the conditional process model. In the mediator model, the linear term on option breadth was positive (*b* = 0.233, *SE* = 0.032, *p* < 0.001) and so was the quadratic term (*b* = 0.198, *SE* = 0.024, *p* < 0.001), which is the convex rise H2 predicted; H2 is supported. The convexity has a substantive corollary: the fitted regret curve has its own minimum at −0.59 standard deviations of option breadth, so that among the most option-poor participants, a wider option set was associated with slightly *less* anticipated regret, while above that point, the association turned positive and steepened.

In the outcome model, anticipated regret was positively associated with difficulties (*b* = 0.330, *SE* = 0.032, *p* < 0.001), supporting the first part of H3, and the direct association of option breadth remained negative (*b* = −0.194, *SE* = 0.030, *p* < 0.001). The result that bears most directly on the mechanism is the quadratic term in the outcome model. Once anticipated regret is in the equation, the squared term on option breadth falls from 0.089 in the reduced form to 0.020 and is no longer distinguishable from zero (*p* = 0.368). Accounting for anticipated regret thus reduced the quadratic coefficient by 78.0% (bootstrap 95% CIs from 5000 resamples: relative reduction [51%, 145%]; absolute reduction 0.069, [0.050, 0.090]). This is a comparison of two coefficients in an observational model rather than a partition of the curvature into causal components, and the interval is wide, as intervals for ratios of coefficients are. The outcome model also contains the regret × self-efficacy product; entering anticipated regret without it gives essentially the same reduction (78.5%). Because anticipated regret and CDD are both self-reported and share some content, the model was also re-estimated separately for each CDDQ cluster with similar reductions (75% to 84%; Appendix A, Table A3); because the clusters were almost perfectly correlated in this sample (Section 4.1), this is a weak check, and the main evidence against construct overlap is the discriminant analysis reported in Section 4.1.

**Figure 4:**
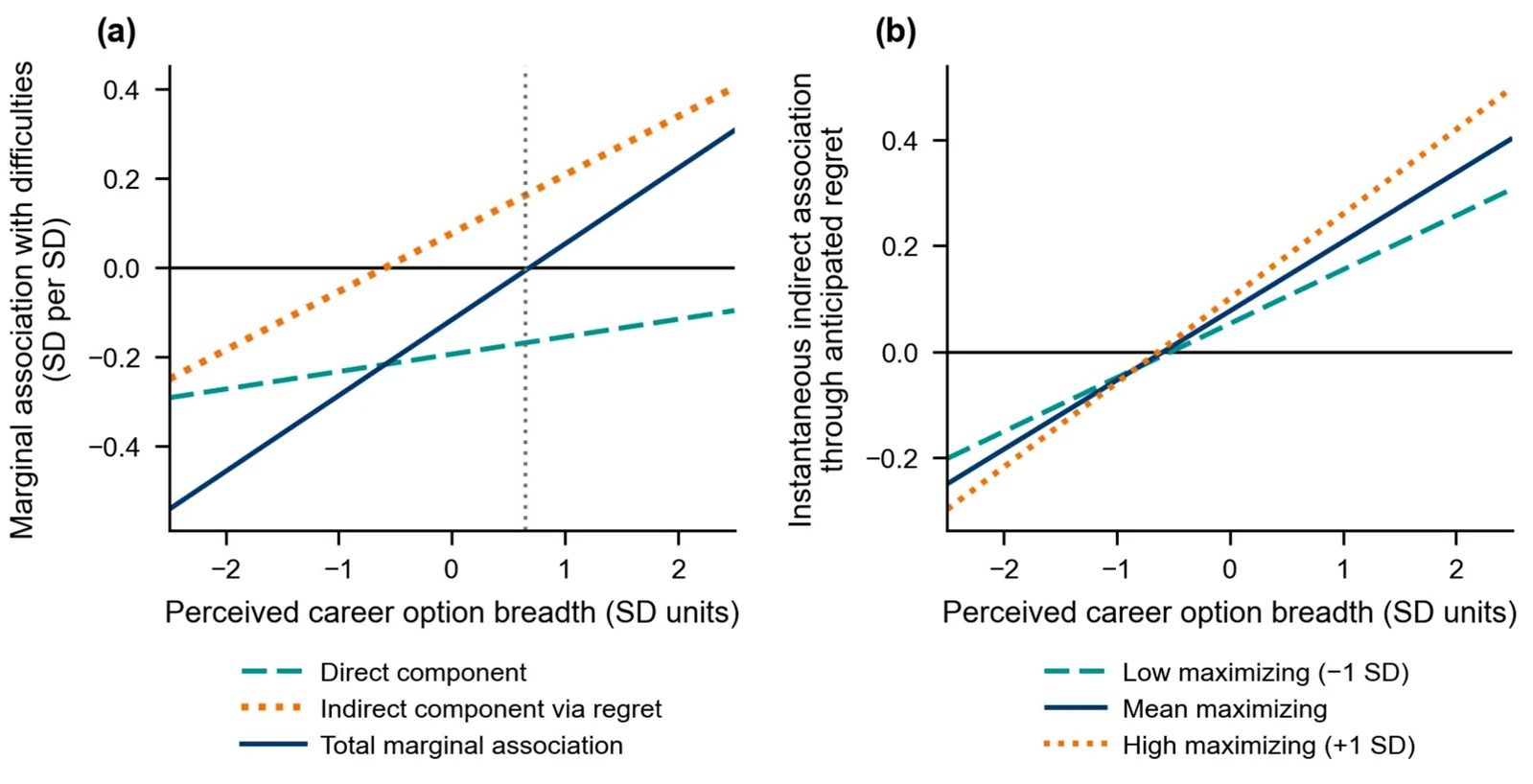
Decomposition of the total marginal association into a direct component and a component carried by anticipated regret. (**a**) The two components and their sum at the mean level of maximizing tendency; (**b**) the regret-carried component at three levels of maximizing tendency. Note. The vertical dotted line in panel (**a**) marks the turning point of the total association. The direct component is the derivative of the direct path in Equation (2); the regret-carried component is the instantaneous indirect association of Equation (6) with self-efficacy at its mean. *N* = 1036.

Because option breadth enters the mediator model quadratically, the indirect association is a function of the predictor rather than a constant (Equation (6)). Table 7 evaluates it at three levels of option breadth and, for each, at three levels of maximizing tendency, holding self-efficacy at its mean. At one standard deviation below the mean of option breadth, the instantaneous indirect association was negative (−0.054, 95% CI [−0.094, −0.016]), meaning that among option-poor participants, the component carried by anticipated regret had the same negative sign as the direct association. At the mean, it had turned positive (0.077, 95% CI [0.053, 0.104]), and at one standard deviation above the mean, it was 0.208 (95% CI [0.157, 0.262]), which is nearly three times its value at the mean. The direct association, in contrast, was negative and close to constant across the same range, moving only from −0.233 to −0.155. H3 is supported. Figure 4 sets the two components side by side: panel (a) shows the near-constant negative direct component, the rising regret-carried component and their sum, which crosses zero at the turning point; panel (b) shows how the regret-carried component steepens with maximizing tendency.

### 4.4. Maximizing Tendency and the Location of the Turning Point

The product of option breadth and maximizing tendency in the mediator model was positive (*b* = 0.072, *SE* = 0.029, *p* = 0.011), as was the product of the squared term and maximizing (*b* = 0.044, *SE* = 0.022, *p* = 0.0497); together, they added 0.009 to the explained variance of anticipated regret. Simple slopes trace what this means. At the mean of option breadth, the association with anticipated regret was 0.161 among low maximizers, 0.233 at the mean, and 0.305 among high maximizers, all with *p* < 0.001; one standard deviation above the mean of option breadth, the corresponding slopes were 0.470, 0.630 and 0.789. The index of moderated mediation was 0.024 with a bootstrap 95% interval of [0.007, 0.044], which excludes zero.

**Figure 5:**
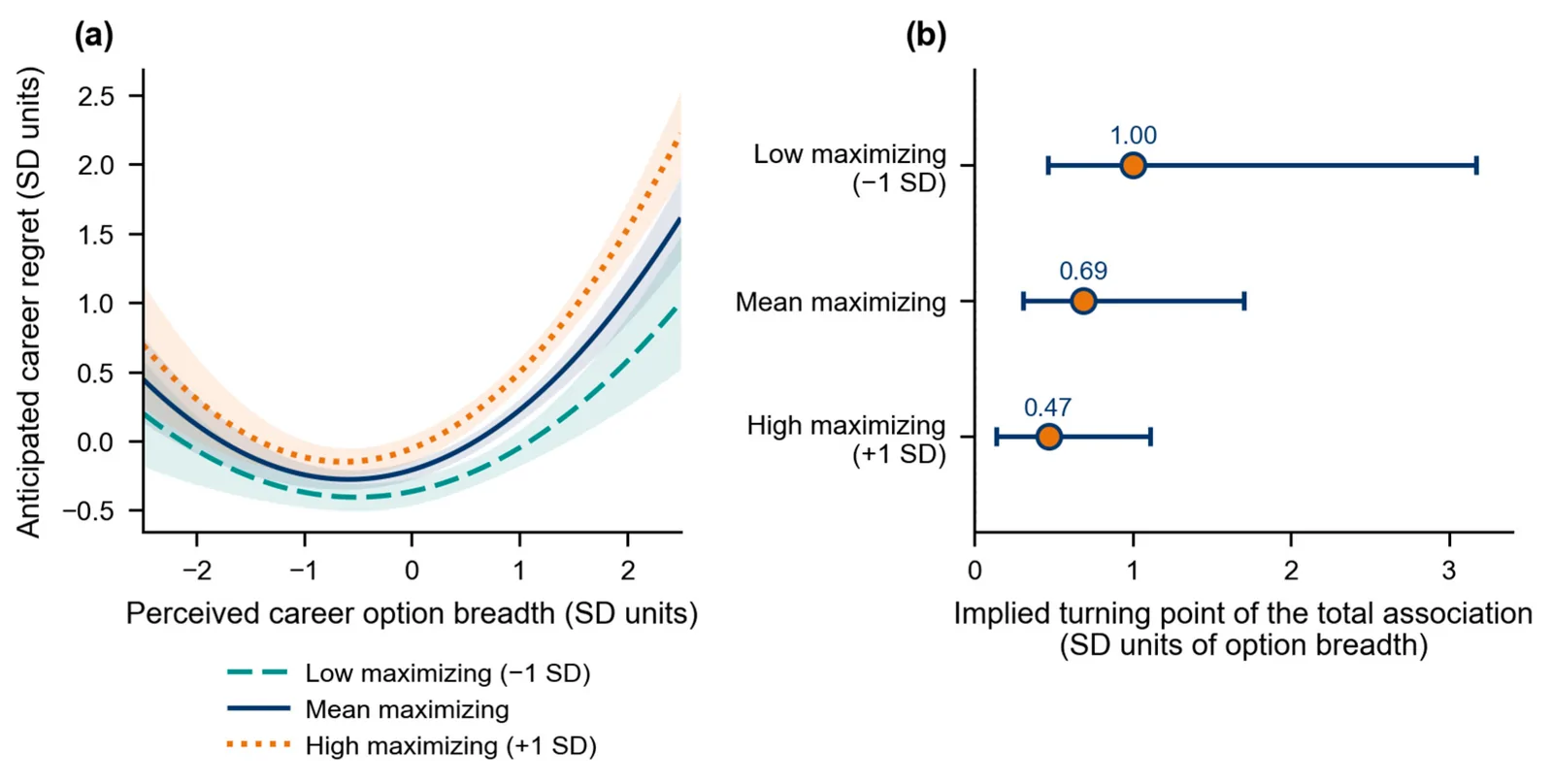
Anticipated career regret and the location of the turning point by maximizing tendency. (**a**) Fitted anticipated career regret as a function of perceived option breadth at three levels of maximizing tendency; (**b**) the implied turning point of the total association at the same three levels. Note. Shaded bands in panel (**a**) are 95% confidence intervals. Points in panel (**b**) are point estimates and horizontal bars are percentile bootstrap 95% intervals from 2000 resamples. Levels of maximizing tendency are one standard deviation below the mean, at the mean, and one standard deviation above. *N* = 1036.

The consequence for the shape of the total association is the part of H4 that matters practically. Setting to zero the total marginal association implied by Equations (1) and (2), that is, the direct component plus Equation (6), gives a turning point of 1.00 standard deviations of option breadth among low maximizers ([0.46, 3.17]), 0.69 at the mean ([0.31, 1.70]) and 0.47 among high maximizers ([0.14, 1.11]). The value at the mean differs slightly from the reduced-form estimate of 0.66 because it is implied by Equations (1) and (2) rather than estimated directly, and the upper limit of the interval for low maximizers lies beyond the observed range. For participants who searched alternatives more exhaustively, the turning point lay at an option breadth roughly half a standard deviation lower than for those who did not. The intervals overlap, so the ordering is better read as a consistent gradient than as three separated estimates, but the index of moderated mediation on which it rests is itself significant. H4 is supported. Figure 5 displays both halves of the result: panel (a) the fitted regret curves at three levels of maximizing tendency, panel (b) the turning points they imply.

### 4.5. Career Decision-Making Self-Efficacy

H5 was not supported. Career decision-making self-efficacy was strongly and negatively associated with career decision-making difficulties (*b* = −0.260, *SE* = 0.028, *p* < 0.001), as the literature would lead one to expect, but its product with anticipated regret fell short of the conventional threshold (*b* = −0.054, *SE* = 0.028, *p* = 0.055, Δ*R*^2^ = 0.003), and the index of moderated mediation for self-efficacy was −0.013 with a bootstrap 95% interval of [−0.0260, 0.0001], which contains zero. When option breadth was scored without item 2, item 4 or both, or from factor scores (estimated with and without the freed residual covariance; Section 3.3), the product ranged from *p* = 0.042 to 0.059 and fell below 0.05 in two of the five scorings, so the test of H5 lies close to the threshold whichever scoring is used, and the null result should not be read as evidence that self-efficacy has no moderating role (Appendix A, Table A1). A Johnson–Neyman analysis (Preacher et al., 2006) found the association between anticipated regret and difficulties to be significantly positive at every level of self-efficacy in the observed range. The sign of the product is the one H5 predicted, and the estimate is close to the threshold, but on this evidence, self-efficacy is better described as a correlate of lower difficulty at every level of anticipated regret than as a condition that changes how strongly regret is associated with difficulty. We treat H5 as unsupported and do not interpret the coefficient further.

### 4.6. Robustness

Table 8 collects the robustness checks on the quadratic term. Removing all covariates, adding city fixed effects, excluding the observations with a Cook’s distance above 4/*n*, Winsorizing the outcome at the first and ninety-ninth percentiles, and estimating the model by median regression all leave the linear and quadratic terms significant with the same signs and a turning point between 0.66 and 0.86 standard deviations. A restricted cubic spline with four knots rejects linearity (*F* = 5.89, *p* = 0.003) and is fitted slightly less well than the quadratic by AIC (2795.0 against 2790.2; the linear model gives 2805.4), so the quadratic is retained as the more parsimonious description. Re-estimating in the count metric returns the turning point of 6.5 options reported above.

Two further sets of checks concern generality. Splitting the sample by status leaves the quadratic term significant among students (*b* = 0.087, *p* = 0.008, *n* = 676) and among early-career workers (*b* = 0.091, *p* = 0.004, *n* = 360), although among workers, the linear term is not significant (−0.056, *p* = 0.274), so the descending arm is less clearly established in that subgroup, and the turning point sits lower. Splitting by gender leaves both terms significant in each group. Estimating the model separately on the three CDDQ clusters returns a significant quadratic term for lack of readiness (*b* = 0.084, *p* < 0.001), lack of information (*b* = 0.081, *p* < 0.001) and inconsistent information (*b* = 0.092, *p* < 0.001), so the pattern is not confined to one component of the outcome, although the clusters were not empirically separable in this sample (Section 4.1). The curvature did not differ significantly between students and early-career workers (joint test of the two interaction terms, *F*(2, 1020) = 0.78, *p* = 0.459) or with age (*F*(2, 1020) = 0.12, *p* = 0.886), and excluding the 13 participants older than 30 left it unchanged (*b* = 0.093, *p* < 0.001). It did vary across cities (*F*(5, 1007) = 3.06, *p* = 0.010): the quadratic term was positive in five of the six cities but individually significant in only two. With 149 to 216 participants per city, these estimates are imprecise, but the variation is a reason not to treat the turning point as fixed across labor markets (Appendix A, Table A3).

Two checks bear directly on the ascending arm. Restricting the sample to the middle 95% of the option-breadth distribution leaves the linear term intact but reduces the quadratic term to 0.031 and renders it non-significant (*p* = 0.375); at a 1% trim, the quadratic term survives (0.071, *p* = 0.015). This is what one should expect. Curvature is identified by the ends of the predictor’s range, and the turning point lies near the seventy-third percentile, so trimming the upper tail removes the observations that carry most of the information about the ascending arm. A two-lines test (Simonsohn, 2018), which estimates separate slopes on either side of a breakpoint without assuming a quadratic form, gives the same message. The descending line was significantly negative under every breakpoint examined. The ascending line was significantly positive when the break was placed at the estimated turning point (*b* = 0.276, 95% CI [0.040, 0.512], *p* = 0.022), but not when the break was set by the Robin Hood algorithm in the flat region near 0.12 standard deviations (*b* = 0.026, *p* = 0.745), nor in the count metric (*p* ≥ 0.14; Appendix A, Table A3). These checks are a real constraint on the claim: the ascending arm rests on the minority of participants who perceive an unusually wide option set, and its slope is estimated less precisely than the descending one, as the widening confidence band on the right of Figure 2 shows. The data therefore support a robust decline in difficulties over the lower part of the range and provide evidence of an upward turn at the high end that is sensitive to restriction of the predictor range.

## 5. Discussion

This study asked whether the perceived breadth of a young adult’s career option set is related to career decision-making difficulties in the way career practice assumes. Across three waves of data from 1036 Chinese young adults, it is not—or at least not everywhere. The association is negative over most of the observed range, which is the pattern a resource account predicts and which a linear model recovers; it flattens near the third quartile, and there is evidence that it turns positive beyond it, although the upward turn rests on the upper tail of the distribution and is less securely established than the decline (Section 4.6). The curvature does not appear to be an artifact of the functional form imposed: it survives a test constructed for the hypothesis rather than for the squared term and is reproduced by a spline that is not constrained to be quadratic, although a two-lines test with a data-driven breakpoint did not detect a significant ascending line (Section 4.6). Accounting for anticipated career regret reduced the quadratic coefficient by about 78%, and the estimated turning point varied with how exhaustively participants searched alternatives.

### 5.1. Two Regions of One Variable

The clearest way to see what the quadratic term adds is to compare it with the model that would ordinarily be reported. Fitted linearly, perceived option breadth carries a coefficient of −0.171 on career decision-making difficulties, and the natural reading is that widening a young person’s option set is helpful with no qualification. The quadratic model suggests that the negative association holds clearly for the lower 61% of the sample and, less certainly, up to the turning point (73%), is close to zero around it, and may be positive in the upper tail. The two accounts are not competing descriptions of the same population; they are descriptions of different regions of one.

This may help to reconcile two bodies of literature that have talked past each other. Career research in constrained labor markets is consistent with the descending arm. Interview accounts from Chinese undergraduates describe difficulty arising not from an overabundance of options but from their near disappearance, with participants narrowing their search prematurely because the perceived option set was too small to sustain exploration (Pan & He, 2026), and structural disadvantage and anxiety about a foreclosed future are associated with poorer perceived work prospects and greater decision difficulty in samples ranging from rural Chinese undergraduates (Wan et al., 2026) to European emerging adults (Pajestka & Paszkowska-Rogacz, 2026). Decision research documents the ascending arm, though almost entirely in consumer settings and almost entirely with assortments the experimenter controls (Iyengar & Lepper, 2000; Chernev et al., 2015; Jacob et al., 2024). The present result places both arms on one axis, in one sample, using a variable that the participant reports rather than the experimenter assigns, although the evidence for the ascending arm is weaker (Section 4.6).

The count metric makes the finding easier to communicate. The fitted minimum lay at about 6.5 occupations under active consideration. The number should not be treated as a general threshold for young people; it is an estimate from one sample drawn from six Chinese cities, obtained with one measurement approach, with a 95% interval of roughly 6 to 8 options; it was obtained from a count that is related to but not interchangeable with the breadth scale; and the mechanism suggests it should shift with the difficulty of comparing the particular options in view. Its order of magnitude is nonetheless informative, although any comparison with the assortment sizes at which choice overload appears in consumer experiments (Chernev et al., 2015) is loose.

### 5.2. Anticipated Regret and the Curvature

The mediation result is the part of this study that points to a candidate mechanism behind the functional form. Anticipated career regret rose with perceived option breadth and rose convexly, as regret regulation theory implies it should when regret is anchored on the forgone alternatives that remain attractive (Zeelenberg & Pieters, 2007). When regret was entered into the outcome equation, the squared term on option breadth fell from 0.089 to 0.020 and lost significance, which was a reduction of roughly 78%. The direct association, meanwhile, stayed negative and nearly constant across the whole range. The pattern is therefore consistent with the sum of a steady negative association and a growing positive one carried by anticipated regret rather than with a single process that reverses; the design does not establish that either component is causal. Because the regret scale has been validated only in this sample, the decomposition should also be treated as provisional.

Decomposing the association this way also explains a pattern that would otherwise look anomalous. The regret pathway had its own minimum at −0.59 standard deviations of option breadth, so among the most option-poor participants, a wider option set was associated with slightly less anticipated regret rather than more. That is consistent with the mechanism: a young adult with almost nothing open to them has little to forgo, and the first additional option is more plausibly experienced as an escape from foreclosure than as a rival to be compared. The cost of forgone alternatives only becomes the operative consideration once there are alternatives worth forgoing.

The finding sits alongside, rather than against, work treating regret as a consequence of career decisions. Regret has mostly been studied retrospectively as something that forms after a decision produces a realized loss (Schulze Schleithoff et al., 2026). The present result concerns its prospective counterpart and suggests that the anticipation may matter before any loss occurs, possibly by making commitment feel expensive.

### 5.3. Who Reaches the Turning Point First

Maximizing tendency behaved as a first-stage condition. The association of option breadth with anticipated regret was steeper among participants who searched alternatives more exhaustively, the interval for the index of moderated mediation excluded zero, and the implied turning point moved from 1.00 standard deviations among low maximizers to 0.47 among high maximizers. The location of the turning point thus appears to be partly a property of the chooser. This is the career-decision analogue of the finding that maximizing job seekers obtain better jobs while feeling worse about them throughout the search (Iyengar et al., 2006), and it is consistent with the measurement work showing that it is the alternative-search component of maximizing, not the high-standards component, that carries the association with regret (Turner et al., 2012; Cheek & Schwartz, 2016).

If confirmed in intervention research, this pattern would be uncomfortable for a service model built on breadth: the same widening of options that is associated with fewer difficulties for one young adult may be associated with more for another, depending on characteristics that are cheap to measure but rarely measured. The intervals around the three turning points overlap, so the gradient should be read as a direction rather than as three distinct values, but the ordering is what the mechanism predicts and the index on which it rests has an interval that excludes zero.

### 5.4. What Self-Efficacy Did and Did Not Do

Career decision-making self-efficacy was, with anticipated regret, one of the two strongest correlates of decision difficulty in the outcome model (*b* = −0.260, *p* < 0.001), which matches the meta-analytic evidence that process-related efficacy is the self-evaluation most closely tied to career indecision (Udayar et al., 2020). It did not, however, moderate the association between anticipated regret and difficulty at the conventional threshold, and the interval for the index of moderated mediation for self-efficacy contained zero. We report this as a null result rather than as a trend. Two readings are available, and the data do not separate them. Self-efficacy may genuinely operate as a level effect, associated with lower difficulty at every level of anticipated regret without changing how strongly regret is associated with difficulty; or the product term may be too small to detect at this sample size: simulation from the fitted model put the power to detect a product term of the observed size (*f*^2^ = 0.004) at 0.51, so the null result does not rule out a small moderating effect of the kind that non-experimental data are poorly placed to detect (McClelland & Judd, 1993). What can be said is that the confidence a young adult has in the decision process was not associated, in this sample, with a weaker link between expecting to regret a choice and difficulty in making it.

### 5.5. Implications for Practice

Three implications follow. They are offered as hypotheses for practice and for intervention research rather than as recommendations, because the study did not manipulate option breadth or test any counseling intervention. The first is diagnostic. If the association between option breadth and difficulty changes sign within the range of ordinary clients, then the size of a client’s live option set may be worth measuring rather than assuming. The single count item used here takes seconds to administer and could indicate roughly where on the fitted curve a client lies, although the turning point estimated here is specific to this sample and measurement approach, and an upward turn was not detected in the count metric itself (Appendix A, Table A3).

The second concerns the content of help. For clients below the turning point, the present associations are consistent with the conventional emphasis on information, exposure and the widening of a set that is too small to choose from. For clients above it, they suggest that further widening may not be associated with lower difficulty, and the mechanism points to work on the forgone alternatives themselves, such as elimination rules, precommitment and the deliberate closing of options, as a candidate for intervention research. Some career decision-making strategies, such as procrastination and the speed of reaching a final decision, are associated with decisional difficulty (Levin et al., 2024), but whether teaching option-closing strategies reduces difficulty among option-rich clients has not been tested.

The third concerns targeting. Because the estimated turning point varies with maximizing tendency, the same option-rich environment, a large career fair or an employer database, may be experienced differently by clients whose search styles differ. Measuring the alternative-search component of maximizing is inexpensive and could help identify clients for whom a wider option set may be associated with more difficulty. Any proposal that different clients should receive different kinds of help, however, assumes that changing option breadth or decision strategies would change decisional difficulty, which this design cannot establish; randomized comparisons of breadth-widening and option-closing interventions, stratified by baseline option breadth and maximizing tendency, are needed before such targeting is adopted.

## 6. Limitations and Future Directions

The first limitation is the one the design cannot remove. Measuring the predictor, the mediator and the outcome on three occasions four weeks apart builds the hypothesized temporal order into the measurement and reduces the most obvious source of consistency artifacts, but it does not identify a causal effect. A young adult who is already struggling to decide may report a wider option set because they have failed to eliminate anything, which would produce the same correlation in reverse. Nor does the design model change. The mediator and the outcome were each measured once, so within-person change cannot be separated from stable differences between persons, which requires repeated measures of the same constructs (Hamaker et al., 2015), and prior levels of anticipated regret and difficulty cannot be controlled, which is an omission that can bias estimates of mediation (Maxwell & Cole, 2007). The decisive test is experimental: assigning participants to consider option sets of different sizes and measuring anticipated regret and decisional difficulty directly. The consumer literature has run that experiment many times (Iyengar & Lepper, 2000; Scheibehenne et al., 2010); the career literature has not, and the present estimates supply the range over which such a manipulation would be informative.

The second concerns measurement. No validated instrument exists for the perceived size of a live career option set, so the scale used here was written for this study. Its internal consistency was good (α = 0.873) and it correlated with the separate count item at *r* = 0.71, but it has not been validated against external criteria in an independent sample, and the two measures may capture partly different things: the scale is closer to a sense of possibility, the count closer to an active consideration set. Anticipated career regret was likewise measured with items written from theory rather than with an established instrument. Both scales were evaluated in the same sample that was used to test the hypotheses, which can make the measurement evidence look more favorable than it would in an independent sample (Clark & Watson, 2019), and the checks reported in Section 4.1 reduce but do not remove the possibility that part of the association between anticipated regret and CDD reflects shared content. Independent validation of both, including cross-validation in new samples (which should also establish whether the residual covariance between option-breadth items 2 and 4 recurs) and tests against external criteria, is a prerequisite for taking the numerical turning point seriously.

The third concerns the region of the data on which the key claim rests. The ascending arm is estimated from the minority of participants who perceive an unusually wide option set, and its confidence band widens accordingly. As reported in Section 4.6, deleting the outer 2.5% of each tail renders the quadratic term non-significant, and a two-lines test with a data-driven breakpoint does not detect a significant ascending line. The trimming result is arithmetically unsurprising, because curvature is identified from the ends of a range, but it means the ascending arm is the less securely established of the two. Oversampling young adults with large consideration sets, or recruiting in settings where they are common, would test it directly.

Fourth, all constructs are self-reported by a single informant. Temporal separation and varied response formats reduce common method variance without eliminating it (Podsakoff et al., 2024), and the diagnostics reported in Section 3.4 are screens rather than proofs. Behavioral indicators of the consideration set, such as the range of positions actually applied for, and informant reports from counselors would provide a stronger test.

Fifth, unmeasured confounding remains possible. Trait indecisiveness, intolerance of uncertainty or ambiguity (Xu & Adams, 2020), neuroticism, perceived employability, socioeconomic resources beyond those recorded, career adaptability and perceived barriers could each be related to perceived option breadth, anticipated regret and decisional difficulty. A sensitivity analysis (Cinelli & Hazlett, 2020) indicates how strong such a confounder would need to be: to render the quadratic term non-significant, it would have to explain at least 6.6% of the residual variance of both the squared option-breadth term and CDD, which is far more than any observed covariate explains of the squared term (at most 0.5%); the corresponding value for the association between anticipated regret and CDD is 23.9% (Appendix A, Table A2). These values bound, but do not rule out, confounding.

Sixth, the sample is specific. It comprises young adults aged 18 to 33 (eligibility 18 to 35) in six Chinese cities during a period of unusually intense graduate competition, and its members were selected on being in the middle of a career decision. Most participants were in their early-to-mid twenties (Section 3.2), and final-year students and early-career workers are at different stages of career development; the curvature did not differ significantly by status or age, but the descending arm was less clearly established among workers (Section 4.6). The recruitment channel of individual participants was not recorded, and the curvature varied across cities. The mechanism is not obviously culture-bound, but the location of the turning point is likely to be: it should move with the difficulty of comparing the options in view, the reversibility of an initial choice and the strength of family expectations about which options are acceptable. Replication in labor markets with different opportunity structures, and in samples of young Chinese workers already in employment (Leong et al., 2025), would establish how far the number travels.

Finally, the model is one specification among several that the theory permits. Maximizing was modeled as a first-stage condition and self-efficacy as a second-stage one, on theoretical grounds, but each could plausibly enter at both stages, and anticipated regret is unlikely to be the only mechanism on the ascending arm; information-processing load and the anticipated difficulty of justifying a choice are obvious rivals. Two further extensions look worthwhile. One is intensive longitudinal measurement across a job-search episode, which would show whether the consideration set contracts as the deadline approaches and whether anticipated regret tracks that contraction within persons. The other is to examine how the growing salience of artificial intelligence in occupational forecasting changes the perceived option set itself, since anxiety about which occupations will still exist is now a measurable correlate of career decisions among students (Duan et al., 2026).

## 7. Conclusions

Among young adults making a career decision in six Chinese cities, the perceived breadth of the career option set showed evidence of a U-shaped rather than a monotone association with career decision-making difficulties. Difficulties were lower at greater breadth up to a fitted minimum 0.66 standard deviations above the mean (about 6.5 occupations in a parallel model using the count of options under active consideration, an estimate specific to this sample and measurement approach), and the upward turn beyond that point was less precisely estimated than the decline. Anticipated career regret rose convexly with option breadth, and accounting for it substantially reduced the quadratic coefficient, while the direct association remained negative throughout; young adults who searched alternatives more exhaustively reached the estimated turning point with a narrower option set. Career decision-making self-efficacy was strongly associated with lower difficulty but did not detectably condition the regret pathway. The practical point is not that choice is bad for young people. It is that the lower difficulty associated with a wider option set appears to be bounded, and whether services can help clients who are past that bound by doing something different is a question for intervention research.

## Figures and Tables

**Table 1:** Characteristics of the analytic sample.

Characteristic	Value
Age (years), *M* (*SD*)	23.69 (3.01)
Gender: women	578 (55.79)
Gender: men	458 (44.21)
Final-year undergraduates	419 (40.44)
Final-year master’s students	257 (24.81)
Early-career workers (≤3 years)	360 (34.75)
Junior-college qualification	145 (14.00)
Bachelor’s degree	576 (55.60)
Master’s degree or above	315 (30.41)
Urban household registration	606 (58.49)
First-generation university entrant	632 (61.00)
Only child	406 (39.19)
City: Hangzhou	216 (20.85)
City: Jiaxing	169 (16.31)
City: Guangzhou	161 (15.54)
City: Tianjin	149 (14.38)
City: Wuhan	177 (17.08)
City: Chengdu	164 (15.83)
Options under active consideration, *M* (*SD*)	4.88 (2.39)
Options under active consideration, median [IQR]	5 [3, 6]

Note. *N* = 1036 young adults who completed all three waves. Percentages are of the analytic sample and may not sum to 100 because of rounding. Gender was recorded as a binary category (woman or man). Options under active consideration is the participant’s own count of distinct occupations being weighed at Wave 1. Source: authors’ calculations from the three-wave survey.

**Table 2:** Descriptive statistics, internal consistency, and zero-order correlations.

Variable	*M*	*SD*	Skew.	Kurt.	α	ω	1	2	3	4	5	6
1. POB (Wave 1)	4.40	1.07	−0.08	−0.18	0.873	0.874	—					
2. MAX (Wave 1)	3.41	0.68	−0.11	−0.28	0.877	0.877	0.143 ***	—				
3. CDSE (Wave 1)	3.50	0.62	−0.14	−0.38	0.956	0.956	0.210 ***	0.021	—			
4. CE (Wave 1)	3.42	0.69	−0.31	0.12	0.861	0.862	0.290 ***	0.094 **	0.243 ***	—		
5. ACR (Wave 2)	4.15	1.03	0.24	−0.09	0.885	0.886	0.275 ***	0.250 ***	0.065 *	0.133 ***	—	
6. CDD (Wave 3)	2.92	0.58	0.14	0.04	0.961	0.961	−0.177 ***	0.132 ***	−0.299 ***	−0.134 ***	0.278 ***	—

Note. *N* = 1036. POB = perceived career option breadth (Wave 1); MAX = maximizing tendency (Wave 1); CDSE = career decision-making self-efficacy (Wave 1); CE = career exploration (Wave 1); ACR = anticipated career regret (Wave 2); CDD = career decision-making difficulties (Wave 3). Means and standard deviations are in the original response metric; correlations are Pearson coefficients. * *p* < 0.05, ** *p* < 0.01, *** *p* < 0.001.

**Table 3:** Convergent and discriminant validity of the measurement model.

Construct	Items	CR	AVE	√AVE	1	2	3	4	5
1. Perceived career option breadth	5	0.874	0.582	0.763	—				
2. Maximizing tendency	7	0.877	0.506	0.711	0.163	—			
3. Career decision-making self-efficacy	25/5	0.959	0.822	0.907	0.230	0.043	—		
4. Anticipated career regret	6	0.886	0.564	0.751	0.312	0.284	0.073	—	
5. Career decision-making difficulties	36/9	0.961	0.732	0.856	0.193	0.144	0.312	0.302	—

Note. *N* = 1036. CR = composite reliability; AVE = average variance extracted. In the Items column, a value of the form 25/5 denotes 25 items entered as 5 parcels. Values below the diagonal in the numbered columns are heterotrait–monotrait (HTMT) ratios of correlations. CR and AVE for career decision-making self-efficacy and for career decision-making difficulties are computed on the parcels used in the measurement model; item-level AVE is reported in the text. Source: authors’ calculations from the three-wave survey.

**Table 4:** Perceived career option breadth and career decision-making difficulties: hierarchical regressions.

Variable	(1)	(2)	(3)	(4)
Perceived career option breadth	—	−0.171 (0.034) ***	−0.162 (0.033) ***	−0.117 (0.032) ***
Option breadth squared	—	—	0.097 (0.026) ***	0.089 (0.024) ***
Maximizing tendency	—	—	—	0.155 (0.029) ***
Career decision-making self-efficacy	—	—	—	−0.257 (0.030) ***
Career exploration	—	—	—	−0.048 (0.031)
Covariates	Yes	Yes	Yes	Yes
Observations	1036	1036	1036	1036
*R* ^2^	0.019	0.047	0.064	0.157
Adjusted *R*^2^	0.011	0.039	0.055	0.146
Δ*R*^2^ vs. previous column	—	0.029	0.017	0.093
*F*	2.62 **	5.29 ***	6.14 ***	13.15 ***

Note. *N* = 1036. The dependent variable is career decision-making difficulties at Wave 3, standardized. Perceived career option breadth is standardized and its square is mean-centered. Entries are unstandardized coefficients with heteroskedasticity-consistent (HC3) standard errors in parentheses. Covariates are age, gender, current status, highest qualification, household registration, first-generation status, family income and only-child status. ** *p* < 0.01, *** *p* < 0.001.

**Table 5:** Testing the U shape: bounds, turning point, and marginal associations.

Quantity	Estimate	*SE*	*t*	*p*	95% CI
Panel A. Test of the composite null in Equation (7)					
Slope at the lowest observed breadth (−2.99 *SD*)	−0.648	0.146	−4.44	<0.001	[−0.934, −0.362]
Slope at the highest observed breadth (2.43 *SD*)	0.315	0.119	2.65	0.004	[0.082, 0.548]
U test statistic (less favorable bound)	—	—	2.65	0.004	—
Turning point (*SD* of option breadth)	0.656	0.249	—	—	[0.276, 1.520]
Turning point (occupational options)	6.53	0.50	—	—	[5.75, 8.05]
Panel B. Marginal association from Equation (4)					
Percentile 10 of option breadth (−1.31 *SD*)	−0.349	0.070	−4.97	<0.001	[−0.487, −0.211]
Percentile 25 of option breadth (−0.75 *SD*)	−0.250	0.048	−5.18	<0.001	[−0.344, −0.155]
Percentile 50 of option breadth (0.00 *SD*)	−0.117	0.032	−3.65	<0.001	[−0.180, −0.054]
Percentile 75 of option breadth (0.75 *SD*)	0.016	0.047	0.34	0.734	[−0.077, 0.109]
Percentile 90 of option breadth (1.31 *SD*)	0.116	0.069	1.67	0.095	[−0.020, 0.251]

Note. *N* = 1036. Panel A implements the test of Equation (7): the composite null of no U shape is rejected only if the slope is significantly negative at the lower bound of the observed data and significantly positive at the upper bound; the reported U statistic is the less favorable of the two one-sided tests (Lind & Mehlum, 2010). *p* values for the two bounds are one-sided. Turning point intervals are Fieller intervals; the corresponding delta-method intervals are [0.17, 1.14] and [5.56, 7.50]. Panel B gives the marginal association of Equation (4) at percentiles of option breadth with two-sided *p* values. All estimates are from column (4) of Table 4, except the turning point in occupational options, which is from the count-metric specification (Section 4.6).

**Table 6:** The conditional process model: anticipated career regret and career decision-making difficulties.

Variable	Anticipated Career Regret (Wave 2)		Career Decision-Making Difficulties (Wave 3)	
	***b* (*SE*)**	***t***	***b* (*SE*)**	***t***
Perceived career option breadth	0.233 (0.032) ***	7.18	−0.194 (0.030) ***	−6.50
Option breadth squared	0.198 (0.024) ***	8.22	0.020 (0.022)	0.90
Maximizing tendency	0.199 (0.028) ***	7.12	0.089 (0.028) **	3.13
Option breadth × maximizing	0.072 (0.029) *	2.53	—	—
Option breadth squared × maximizing	0.044 (0.022) *	1.96	—	—
Anticipated career regret	—	—	0.330 (0.032) ***	10.40
Career decision-making self-efficacy	0.005 (0.029)	0.15	−0.260 (0.028) ***	−9.30
Anticipated regret × self-efficacy	—	—	−0.054 (0.028)	−1.92
Career exploration	0.059 (0.030)	1.95	−0.068 (0.029) *	−2.29
Covariates	Yes		Yes	
Observations	1036		1036	
*R* ^2^	0.225		0.244	
*F*	22.11 ***		20.85 ***	

Note. *N* = 1036. Entries are unstandardized coefficients with heteroskedasticity-consistent (HC3) standard errors in parentheses and *t* statistics in the adjacent column. All variables entering products were standardized before the products were formed. Covariates as in Table 4. Δ*R*^2^ for the two maximizing product terms in the mediator model is 0.009; for the regret × self-efficacy product in the outcome model it is 0.003. * *p* < 0.05, ** *p* < 0.01, *** *p* < 0.001.

**Table 7:** Instantaneous indirect and direct associations of option breadth with career decision-making difficulties.

Option Breadth	Maximizing Tendency	Instantaneous Indirect Association	Bootstrap *SE*	95% CI
Panel A. Instantaneous indirect association				
−1 *SD*	−1 *SD*	−0.049	0.024	[−0.101, −0.006]
−1 *SD*	Mean	−0.054	0.019	[−0.094, −0.016]
−1 *SD*	+1 *SD*	−0.059	0.027	[−0.111, −0.005]
Mean	−1 *SD*	0.053	0.015	[0.024, 0.083]
Mean	Mean	0.077	0.013	[0.053, 0.104]
Mean	+1 *SD*	0.101	0.017	[0.070, 0.137]
+1 *SD*	−1 *SD*	0.155	0.033	[0.094, 0.223]
+1 *SD*	Mean	0.208	0.027	[0.157, 0.262]
+1 *SD*	+1 *SD*	0.260	0.032	[0.202, 0.325]
Panel B. Direct association				
−1 *SD*	—	−0.233	—	[−0.327, −0.136]
Mean	—	−0.194	—	[−0.252, −0.137]
+1 *SD*	—	−0.155	—	[−0.266, −0.048]
Panel C. Index of moderated mediation				
Maximizing tendency	—	0.024	—	[0.007, 0.044]
Career decision-making self-efficacy	—	−0.013	—	[−0.0260, 0.0001]

Note. *N* = 1036. Entries are the instantaneous indirect association of Equation (6) evaluated at the stated levels with career decision-making self-efficacy held at its mean; levels are expressed in standard deviations from the mean. Confidence intervals are percentile intervals from 5000 bootstrap resamples. The direct association of Equation (2) and the index of moderated mediation are reported in Panels B and C, respectively. Source: authors’ calculations from the three-wave survey.

**Table 8:** Robustness of the quadratic association.

Specification	*n*	Linear Term	Quadratic Term	Turning Point
Baseline (Table 4, column 4)	1036	−0.117 (0.032) ***	0.089 (0.024) ***	0.66
No covariates	1036	−0.169 (0.032) ***	0.098 (0.026) ***	0.86
City fixed effects	1036	−0.118 (0.032) ***	0.088 (0.023) ***	0.67
Excluding influential observations (Cook’s *D* > 4/*n*)	979	−0.120 (0.027) ***	0.072 (0.020) ***	0.83
Outcome Winsorized at 1% and 99%	1036	−0.113 (0.031) ***	0.084 (0.022) ***	0.67
Median (quantile) regression	1036	−0.131 (0.038) ***	0.100 (0.027) ***	0.66
Option count in place of the scale	1036	−0.2325 (0.0426) ***	0.0178 (0.0040) ***	6.53 options
Trimming 1% from each tail of option breadth	1010	−0.112 (0.034) ***	0.071 (0.029) *	0.79
Trimming 2.5% from each tail of option breadth	976	−0.139 (0.036) ***	0.031 (0.035)	2.22
Final-year students only	676	−0.146 (0.041) ***	0.087 (0.033) **	0.84
Early-career workers only	360	−0.056 (0.051)	0.091 (0.032) **	0.31
Women only	578	−0.098 (0.046) *	0.080 (0.036) *	0.61
Men only	458	−0.131 (0.046) **	0.101 (0.032) **	0.65
Outcome: lack of readiness	1036	−0.104 (0.032) **	0.084 (0.023) ***	0.62
Outcome: lack of information	1036	−0.132 (0.032) ***	0.081 (0.024) ***	0.82
Outcome: inconsistent information	1036	−0.102 (0.032) **	0.092 (0.024) ***	0.55

Note. The dependent variable is career decision-making difficulties at Wave 3. Entries are unstandardized coefficients with heteroskedasticity-consistent (HC3) standard errors in parentheses, except for the median regression, which reports kernel-based robust standard errors. Unless stated otherwise, the specification is column (4) of Table 4. The turning point is in standard deviations of option breadth, except in the count-metric row, where it is a number of occupations. Trimming rows delete the stated percentage from each tail of the option-breadth distribution and are reported for transparency; they show how far the quadratic term depends on the tails of the distribution (Section 4.6). * *p* < 0.05, ** *p* < 0.01, *** *p* < 0.001.

## Data Availability

The data presented in this study are available on request from the corresponding author. They are not publicly available because participants did not consent to open deposition of individual-level responses. The analysis code that reproduces every table and figure is available from the corresponding author on request.

## Abbreviations

The following abbreviations are used in this manuscript:

Abbreviation	Definition
ACR	Anticipated career regret
AIC	Akaike information criterion
AVE	Average variance extracted
CDD	Career decision-making difficulties
CDDQ	Career Decision-Making Difficulties Questionnaire
CDSE	Career decision-making self-efficacy
CE	Career exploration
CFA	Confirmatory factor analysis
CFI	Comparative fit index
CI	Confidence interval
CR	Composite reliability
EFA	Exploratory factor analysis
GFI	Goodness-of-fit index
HC3	Heteroskedasticity-consistent covariance estimator, third variant
HTMT	Heterotrait–monotrait ratio of correlations
IQR	Interquartile range
MAX	Maximizing tendency (alternative-search component)
POB	Perceived career option breadth
RMSEA	Root mean square error of approximation
SRMR	Standardized root mean square residual
TLI	Tucker–Lewis index

## Appendix A

This appendix reports additional psychometric, power and robustness analyses. Table A1 collects the psychometric checks on the measurement model and on the two scales written for this study, Table A2 the power and sensitivity analyses, and Table A3 the additional robustness and heterogeneity analyses. Panel A of Table A1 also repeats the hypothesis tests with alternative scores for option breadth. All estimates use the analytic sample of 1036 unless stated otherwise; the analysis script is available from the corresponding author with the other code.

**Table A1:** Additional psychometric evidence.

Analysis	*n*	χ^2^ (*df*)	CFI/TLI/RMSEA/SRMR	Key Estimates
Panel A. Perceived career option breadth (POB)				
One-factor CFA, full sample	1036	63.43 (5)	0.976/0.952/0.106/0.027	λ = 0.69–0.80
One-factor CFA, residual covariance of items 2 and 4 freed	1036	1.77 (4)	1.000/1.000/0.000/0.005	largest residual *r* in the one-factor model = 0.06; λ = 0.71–0.83; correlation between the unique factors of items 2 and 4 = 0.31; ω = 0.852 with their covariance counted as error variance
EFA, random half A	518	—	—	1 factor retained (parallel analysis); first eigenvalue 3.22 (64.4% of total variance); λ = 0.66–0.80
One-factor CFA, random half B	518	43.51 (5)	0.971/0.942/0.122/0.030	λ = 0.72–0.82
One-factor CFA, residual covariance of items 2 and 4 freed, random half B	518	4.03 (4)	1.000/1.000/0.004/0.010	residual *r*(2, 4) in the one-factor model = 0.064 in half A (largest) and 0.060 in half B (second largest, after *r*(1, 5) = 0.063)
Residual *r*(2, 4) in the one-factor model, 1000 further random splits	518 per half	—	—	positive in all 2000 halves; median 0.061 (2.5th–97.5th percentile 0.043–0.082); largest residual in 92% of halves
Residual *r*(2, 4) in the one-factor model, subgroups by city, career status, gender and education	145–578	—	—	positive in all 14 subgroups (0.03–0.11); largest residual in 8
One-factor CFA of the four-item versions	1036	—	—	RMSEA = 0.000 without item 2 (χ^2^(2) = 0.60) or item 4 (χ^2^(2) = 1.56); 0.100–0.144 without item 1, 3 or 5
H1 to H3 with item 2, item 4 or both removed, or with factor scores in place of the item mean	1036	—	—	CDD quadratic *b* = 0.074 to 0.088 (*p* ≤ 0.002); Lind–Mehlum *p* = 0.005–0.014; turning point 0.62–0.68 standard deviations above the mean (reported model 0.66); ACR quadratic *b* = 0.173 to 0.201 (*p* < 0.001; reported model 0.198); CDD quadratic term reduced by 78–82% when ACR is added (reported model 78%)
H4 and H5 with the same alternative scores	1036	—	—	maximizing product terms jointly *p* = 0.002–0.006; squared term × maximizing *b* = 0.042 to 0.045, *p* = 0.032–0.058 (reported model 0.044, *p* = 0.0497); index of moderated mediation (maximizing) 0.023–0.026, 95% interval excluding zero in all 5; ACR × CDSE *b* = −0.057 to −0.053, *p* = 0.042–0.059 (reported model −0.054, *p* = 0.055); index of moderated mediation (self-efficacy) 95% interval excluding zero in 2 of 5
Panel B. Anticipated career regret (ACR)				
One-factor CFA, full sample	1036	55.91 (9)	0.984/0.974/0.071/0.023	λ = 0.70–0.81
EFA, random half A	518	—	—	1 factor retained; first eigenvalue 3.92 (65.3% of total variance); λ = 0.71–0.80
One-factor CFA, random half B	518	26.17 (9)	0.987/0.979/0.061/0.025	λ = 0.65–0.82
Panel C. Discriminant validity of the new scales				
EFA of POB, CE and ACR items, half A (oblimin)	518	—	—	3 factors retained; own loadings 0.66–0.82; largest cross-loading 0.07
Three-factor CFA (POB, CE, ACR), half B	518	194.54 (116)	0.981/0.977/0.036/0.031	*r*(POB, CE) = 0.30; *r*(POB, ACR) = 0.35; *r*(CE, ACR) = 0.17
POB and CE: two factors vs. one	1036	Δχ^2^ = 1982.37 (1) ***	0.980/0.974/0.047/0.023	latent *r* = 0.33; HTMT = 0.33
ACR and CDD (nine parcels): two factors vs. one	1036	Δχ^2^ = 2664.72 (1) ***	0.993/0.991/0.031/0.020	latent *r* = 0.30; HTMT = 0.30 (0.29–0.31 with each cluster)
ACR and MAX: two factors vs. one	1036	Δχ^2^ = 2575.83 (1) ***	0.982/0.978/0.040/0.024	latent *r* = 0.28
Panel D. Five-factor measurement model				
Sequential parcels (model reported in Section 4.1)	1036	667.92 (454)	0.991/0.990/0.021/0.021	—
Interleaved parcels	1036	1067.13 (454)	0.971/0.969/0.036/0.023	—
All 79 items as indicators (no parcels)	1036	4203.26 (2992)	0.969/0.968/0.020/0.026	λ = 0.49–0.81
CDSE domains and CDDQ clusters as separate first-order factors	1036	4145.22 (2947)	0.969/0.968/0.020/0.026	latent *r* among domains 1.01–1.03 and among clusters 1.00–1.01 (not separable); vs. one factor per instrument, Δχ^2^ = 58.04 (45), *p* = 0.092

Note. CFA = confirmatory factor analysis; EFA = exploratory factor analysis (maximum likelihood); λ = standardized loadings; CE = career exploration; MAX = maximizing tendency; CDD = career decision-making difficulties; HTMT = heterotrait–monotrait ratio. The random halves were drawn once with a fixed seed. Residual *r* is the observed minus the model-implied correlation in the one-factor model; largest refers to absolute value. The 1000 further random splits in Panel A were drawn with a second fixed seed, and the intervals for the indices of moderated mediation are percentile intervals from 5000 bootstrap resamples. Fit indices in rows comparing two factors with one refer to the two-factor model; the Δχ^2^ compares it with the one-factor model. CFI and TLI values above 1 are shown as 1.000. Interleaved parcels assign every fifth CDSE item and every ninth CDDQ item to the same parcel. *** *p* < 0.001.

**Table A2:** Power, sensitivity and robustness to unmeasured confounding.

Test	Numerator *df*/Predictors	Observed Δ*R*^2^ (*f*^2^)	Minimum Detectable *f*^2^ (80% Power)	Power for Observed Effect (Analytic; Simulated)
Panel A. Power and sensitivity				
H1: quadratic term, Equation (3)	1/13	0.014 (0.017)	0.0076	0.99; 0.98
H1: Lind–Mehlum test	—	—	—	—; 0.90
H2: quadratic term, Equation (1)	1/15	0.067 (0.087)	0.0076	>0.99; —
H3: anticipated regret, Equation (2)	1/15	0.085 (0.112)	0.0076	>0.99; —
H4: two product terms, Equation (1)	2/15	0.009 (0.011)	0.0093	0.87; 0.86
H5: product term, Equation (2)	1/15	0.003 (0.004)	0.0076	0.51; 0.51
Panel B. Sensitivity to unmeasured confounding	*t*	Partial *R*^2^ with outcome	Robustness value	Robustness value (α = 0.05)
Quadratic term, Equation (3)	4.14	0.016	0.121	0.066
Quadratic term, Equation (1)	9.40	0.080	0.254	0.207
Anticipated regret, Equation (2)	10.71	0.101	0.284	0.239
Benchmark: largest partial *R*^2^ of an observed covariate	—	0.066 (CDSE, with CDD)	0.005 (CE, with squared POB)	—

Note. Panel A: analytic power and minimum detectable effects follow G*Power’s fixed-model *R*^2^ increase test with λ = *f*^2^*N* and α = 0.05. Simulated power is the share of 2000 samples, generated from the fitted model with resampled residuals and the observed design matrix, in which the test was significant with HC3 standard errors. Panel B: *t* statistics are from ordinary least squares; the robustness value is the partial *R*^2^ that an unobserved confounder would need with both the focal term and the outcome to reduce the estimate to zero, or, in the last column, to render it non-significant at α = 0.05 (Cinelli & Hazlett, 2020). The benchmark row gives the largest partial *R*^2^ of any observed covariate with the outcome (career decision-making self-efficacy) and with the squared option-breadth term (career exploration).

**Table A3:** Additional robustness and heterogeneity analyses.

Analysis	*n*	Estimate	95% CI	*p*
Panel A. Seven or more options versus fewer				
7+ vs. 0–6 options, unadjusted (Cohen’s *d*)	243/793	−0.188	[−0.33, −0.04]	0.012
7+ vs. 0–6 options, adjusted for covariates (*b*, *SD* units)	1036	−0.090	[−0.231, 0.050]	0.208
7+ vs. 5–6 options, unadjusted (Cohen’s *d*)	243/330	−0.024	[−0.19, 0.14]	0.779
7+ vs. 5–6 options, adjusted for covariates (*b*, *SD* units)	573	0.022	[−0.140, 0.184]	0.793
Panel B. The ascending arm				
Region where the slope is significantly negative	629	POB ≤ 0.28 *SD*	—	—
Region where the slope is significantly positive	62	POB ≥ 1.52 *SD*	—	—
Slope at the 95th percentile (1.68 *SD*)	1036	0.182	[0.015, 0.350]	0.033
Two lines, Robin Hood break at 0.12 *SD*: left line	563	−0.284	[−0.421, −0.147]	<0.001
Two lines, Robin Hood break at 0.12 *SD*: right line	473	0.026	[−0.132, 0.184]	0.745
Two lines, break at the turning point (0.66 *SD*): left line	755	−0.182	[−0.285, −0.079]	<0.001
Two lines, break at the turning point (0.66 *SD*): right line	281	0.276	[0.040, 0.512]	0.022
Two lines, count metric, Robin Hood break at 5.47: left line	621	−0.125	[−0.176, −0.073]	<0.001
Two lines, count metric, Robin Hood break at 5.47: right line	415	0.050	[−0.017, 0.118]	0.145
Two lines, count metric, break at 6.53: right line	243	0.065	[−0.033, 0.164]	0.194
Panel C. Mediation re-estimated for each CDDQ cluster				
Lack of readiness: reduction in the quadratic coefficient	1036	76.2%	[50%, 150%]	—
Lack of readiness: indirect association at +1 *SD* of POB	1036	0.193	[0.143, 0.248]	—
Lack of information: reduction in the quadratic coefficient	1036	84.0%	[54%, 180%]	—
Lack of information: indirect association at +1 *SD* of POB	1036	0.203	[0.152, 0.259]	—
Inconsistent information: reduction in the quadratic coefficient	1036	74.6%	[49%, 144%]	—
Inconsistent information: indirect association at +1 *SD* of POB	1036	0.205	[0.154, 0.259]	—
Panel D. Heterogeneity				
City × (POB, POB^2^), joint test	1036	*F*(10, 1007) = 2.26	—	0.013
City × POB^2^, joint test	1036	*F*(5, 1007) = 3.06	—	0.010
Status (worker) × (POB, POB^2^), joint test	1036	*F*(2, 1020) = 0.78	—	0.459
Age × (POB, POB^2^), joint test	1036	*F*(2, 1020) = 0.12	—	0.886
Participants aged 30 or younger: quadratic term	1023	0.093	—	<0.001
Hangzhou: quadratic term	216	0.067	[−0.023, 0.157]	0.143
Jiaxing: quadratic term	169	−0.046	[−0.143, 0.050]	0.345
Guangzhou: quadratic term	161	0.043	[−0.104, 0.190]	0.565
Tianjin: quadratic term	149	0.243	[0.139, 0.348]	<0.001
Wuhan: quadratic term	177	0.093	[−0.036, 0.222]	0.159
Chengdu: quadratic term	164	0.164	[0.056, 0.271]	0.003

Note. Covariates are those of column (4) of Table 4. Panel A compares participants weighing seven or more occupations with those weighing six or fewer (all) or five to six (those near the fitted minimum). Panel B: regions of significance are where the 95% confidence interval of the marginal slope from Equation (4) excludes zero; two-lines tests follow Simonsohn (2018) with the flat region defined from a four-knot restricted cubic spline and HC3 standard errors. Panel C: the reduction is the relative change in the quadratic coefficient when anticipated regret is added; intervals are percentile bootstrap intervals from 2000 resamples. Panel D: tests add the stated interaction terms to column (4) of Table 4 (with city fixed effects for the city tests).
